# ERK1/2 signalling protects against apoptosis following endoplasmic reticulum stress but cannot provide long-term protection against BAX/BAK-independent cell death

**DOI:** 10.1371/journal.pone.0184907

**Published:** 2017-09-20

**Authors:** Nicola J. Darling, Kathryn Balmanno, Simon J. Cook

**Affiliations:** Signalling Laboratory, The Babraham Institute, Babraham Research Campus, Cambridge, CB22 3AT, United Kingdom; Wayne State University, UNITED STATES

## Abstract

Disruption of protein folding in the endoplasmic reticulum (ER) causes ER stress. Activation of the unfolded protein response (UPR) acts to restore protein homeostasis or, if ER stress is severe or persistent, drive apoptosis, which is thought to proceed through the cell intrinsic, mitochondrial pathway. Indeed, cells that lack the key executioner proteins BAX and BAK are protected from ER stress-induced apoptosis. Here we show that chronic ER stress causes the progressive inhibition of the extracellular signal-regulated kinase (ERK1/2) signalling pathway. This is causally related to ER stress since reactivation of ERK1/2 can protect cells from ER stress-induced apoptosis whilst ERK1/2 pathway inhibition sensitises cells to ER stress. Furthermore, cancer cell lines harbouring constitutively active BRAF^V600E^ are addicted to ERK1/2 signalling for protection against ER stress-induced cell death. ERK1/2 signalling normally represses the pro-death proteins BIM, BMF and PUMA and it has been proposed that ER stress induces BIM-dependent cell death. We found no evidence that ER stress increased the expression of these proteins; furthermore, BIM was not required for ER stress-induced death. Rather, ER stress caused the PERK-dependent inhibition of cap-dependent mRNA translation and the progressive loss of pro-survival proteins including BCL2, BCL_XL_ and MCL1. Despite these observations, neither ERK1/2 activation nor loss of BAX/BAK could confer long-term clonogenic survival to cells exposed to ER stress. Thus, ER stress induces cell death by at least two biochemically and genetically distinct pathways: a classical BAX/BAK-dependent apoptotic response that can be inhibited by ERK1/2 signalling and an alternative ERK1/2- and BAX/BAK-independent cell death pathway.

## Introduction

The endoplasmic reticulum (ER) is the site of modification, folding and maturation of transmembrane and secreted proteins and, as an intracellular Ca^2+^ store, plays a prominent role in signal transduction. Increased demand for transmembrane and secreted proteins or perturbations within the ER (e.g., reduced luminal [Ca^2+^] or altered redox status) undermines protein processing in the ER resulting in the accumulation of misfolded proteins [1]. Such ‘ER stress’ elicits the ‘unfolded protein response’ (UPR) which acts to restore ER protein homeostasis by shutting down general protein synthesis, cleaving mRNAs encoding membrane and secretory proteins that would normally be trafficked through the ER and selectively driving the expression of chaperones such as BiP/GRP78 to enhance the protein folding capacity of the ER [2].

The UPR involves three key signalling cascades that are controlled by inositol requiring protein 1 (IRE1), protein kinase R-like ER kinase (PERK) and activating transcription factor 6 (ATF6). PERK and IRE1 span the ER membrane, and contain a luminal domain that detects misfolded polypeptides [3] to initiate signalling through their cytosolic domains. The cytosolic kinase domain of PERK phosphorylates eukaryotic translation initiation factor 2α (eIF2α) thereby inhibiting cap-dependent translation [4]; however, alternative translation initiation mechanisms allow the continued synthesis of a subset of proteins such as the transcription factor ATF4 [5–8] which in turn drives the expression of stress-responsive genes including the transcription factor CCAAT/enhancer-binding protein homologous protein (CHOP) [5]. IRE1 has both cytosolic protein kinase and endoribonuclease (RNase) domains; the kinase domain activates the JNK signalling pathway [9], whereas the RNase domain promotes destruction of ER-associated mRNAs through regulated IRE1-dependent decay (RIDD) [10, 11] and also promotes a non-canonical splicing event to generate the spliced form of transcription factor X-box binding protein 1 (XBP1s) [12]. ATF6 is constitutively expressed in a latent form but following ER stress is processed at the Golgi into an active form that translocates to the nucleus to drive transcription [13].

If ER stress is too severe or persistent, including in certain pathological conditions, UPR signalling can also drive apoptotic cell death [14]. Apoptosis is initiated through two major pathways: the cell intrinsic, mitochondrial pathway, regulated by the BCL2 protein family and the cell extrinsic, death-receptor pathway [15]; each pathway ultimately converges to activate the executioner caspases such as caspase-3. There remains considerable debate about how UPR signalling engages with these core apoptotic pathways. IRE1-dependent de-repression of caspase-2 was proposed [16] but caspase-2 is not required for ER stress-induced apoptosis [17, 18]. In contrast, there is a prominent role for CHOP since CHOP^-/-^ cells are protected from ER stress [19, 20]. Other studies have suggested that ER stress initiates apoptosis via the intrinsic BCL2 pathway [14, 21]; indeed, it has been proposed that ER stress drives apoptosis through the upregulation of the pro-apoptotic BH3-only protein BIM [22, 23], a response that is mediated in part by CHOP. However, it has also been proposed that ER stress drives apoptosis through the CHOP-dependent cell autonomous up-regulation and ligand-independent activation of the death receptor DR5 [17, 24]; how this relates to the BCL2 pathway is unclear.

Here we have investigated ER stress-induced death. We find that ER stress-induces BAX/BAK-dependent apoptosis that can be rescued by ERK1/2 activation but we find no evidence of a role for BIM; rather ER stress causes the PERK-dependent inhibition of mRNA translation and loss of multiple pro-survival BCL2 proteins. Despite this, loss of BAX/BAK or strong ERK1/2 activation fails to confer long-term survival following ER stress. Thus, ER stress induces cell death by at least two alternative pathways: a BAX/BAK-dependent apoptotic response that can be inhibited by ERK1/2 signalling and an alternative, ERK1/2- and BAX/BAK-independent cell death pathway.

## Materials and methods

### Materials

Thapsigargin was purchased from Invitrogen. Tunicamycin was purchased from Enzo Life Sciences. 4-hydroxytamoxifen (4-HT) was purchased from Sigma; in all experiments involving 4-HT 0.1% ethanol, the diluent for 4-HT, was used and was without effect. Selumetinib (AZD6244/ARRY-142886) and AZD8055 were provided by Paul Smith, AstraZeneca, Alderley Park, Macclesfield, UK, whilst SCH772984 was purchased from Selleck Chemicals. ABT-263 was purchased from Santa Cruz Biotechnology. GSK2606414 and QVD-oPh were purchased from MERCK and Calbiochem respectively. Antibodies specific for BCL_XL_ (2762), BiP (3177), CHOP (2895), eIF2α (9722), p-eIF2α (Ser51) (9721), p-ERK1/2 (Thr202/Tyr204) (9106), IRE1α (3294), PARP (9542) and PERK (3192) were purchased from Cell Signaling Technology; ERK1 (610031) from BD Biosciences; BIM (AB17003) from Millipore; BMF (ALX-804-343) from Enzo Life Sciences; PUMA (3043) from ProSci; BAK (sc-832), BAX (sc-493), BCL2 (sc-7382) and MCL1 (sc-819) from Santa Cruz Biotechnology; Atg5 (A0731) and β-Actin (A544) from Sigma. Horseradish peroxidase-conjugated secondary antibodies were from Bio-Rad and the enhanced chemiluminescence (ECL) system from GE Healthcare was used for detection.

### Preparation of cell extracts and Western blotting

Cells were lysed in ice-cold TG lysis buffer, assayed for protein content and analysed by Western blotting following fractionation by SDS-PAGE [25].

### Flow cytometry

Propidium iodide staining and flow cytometry was used to determine the distribution of cells in G1, S, G2/M and dead cells (sub-G1) [26].

### RNA interference

Transient siRNA knockdown of BIM in COLO205 cells was performed as described previously [27]. PERK siRNA (M-004883) and non-targeting siRNA (D-001206) were purchased from Dharmacon. HCT116 cells were transfected using DharmaFECT solution 2 (Dharmacon) and 10 nM siRNA as per manufacturer’s instructions.

### Colony formation assays

Cells were seeded at 200 cells per well in 12-well plates and left to settle for 24 h prior to treatment as indicated. The medium was replaced with fresh medium each week during the assay, cells were allowed to grow for 9–11 days including the treatment period. Subsequently cells were fixed in 75% (v/v) methanol, 25% (v/v) acetic acid and stained with crystal violet. Colonies were assessed either by measuring crystal violet absorbance following solubilisation in 10% (v/v) acetic acid or by counting.

### Cell lines

COLO205 and HT29 cells were purchased from ATCC (Manassas, VA, USA). HCT116 cells and isogenic derivatives were provided by Bert Vogelstein, John Hopkins University and Richard Yule, National Institute of Health, Bethesda. Atg5 WT and Atg5^-/-^ immortalised mouse embryonic fibroblasts (iMEFs) were provided by Noboru Mizushima, Tokyo Medical and Dental University. NIH3T3 ΔCRAF:ER cells [28] were provided by Martin McMahon, University of California, San Francisco. Generation of CCL39 ΔCRAF:ER cells was described previously [29]. Cells were cultured in medium comprising RPMI 1640 (COLO205 cells), McCoy’s 5A (HT29 cells), Dulbecco’s modified Eagle’s medium (HCT116, iMEF and HEK293 cells) or Dulbecco’s modified Eagle’s medium without phenol red (NIH3T3 and CCL39 cells expressing ΔCRAF:ER*) supplemented with glucose (4.5 mg ml^-1^), penicillin (100 units ml^-1^), streptomycin (100 μg ml^-1^), L-glutamine (1 mM), fetal bovine serum (10% v/v), puromycin (6 μg ml^-1^; ΔCRAF:ER cell lines), blasticidin (5 μg ml^-1^; HEK293 TetR cell lines) and zeocin (100 μg ml^-1^; HEK293 TetR EV and CHOP cell lines).

### Generation of HEK293 TetR cell lines

HEK293 TetR cells were provided by Dr Anne Ashford, The Babraham Institute and were generated by transfection of HEK293 cells with pcDNA6/TR (Invitrogen). Plasmid DNA was combined with 13.6 nM CaCl_2_ in HEPES-buffered saline and added dropwise to cells. Cells were selected using 5 μg ml^-1^ blasticidin and were isolated from single cell clones. HEK293 TetR cells were then transfected with pcDNA4/TO (Invitrogen) or pcDNA4/TO Myc-CHOP (human) and stably transfected cells were selected from single cell clones using 100 μg ml^-1^ zeocin.

## Results

### ERK1/2 signalling protects cells from ER stress-induced apoptosis

**Fig 1:**
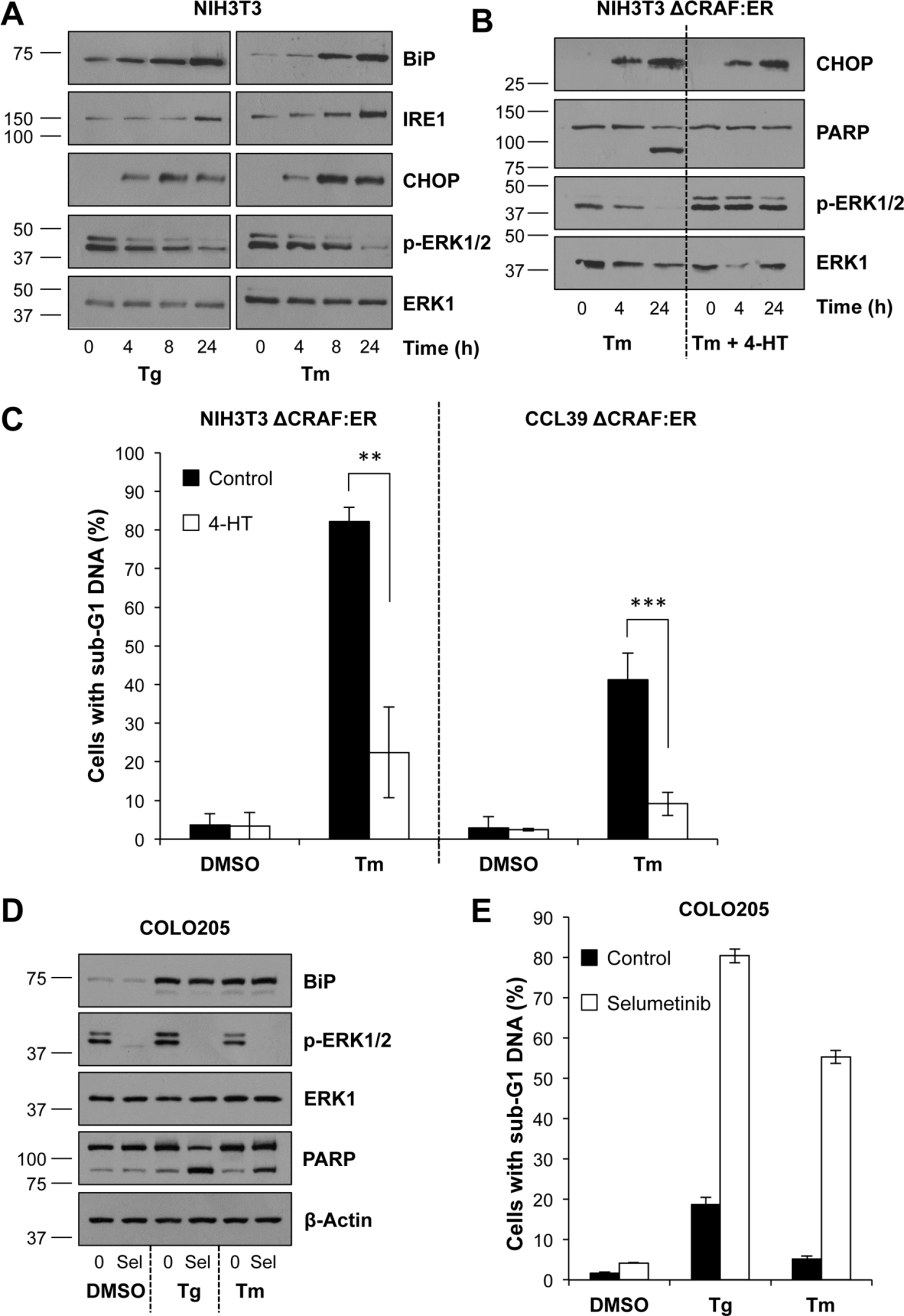
Activation of ERK1/2 protects against ER stress-induced cell death. **(A)** NIH3T3 cells were treated with either 100 nM Tg (left panel) or 2 μg ml^-1^ Tm (right panel) for the indicated time. Whole cell lysates were separated by SDS-PAGE and analysed by immunoblotting using the indicated antibodies. **(B)** NIH3T3 ΔCRAF:ER cells were pre-treated for 1 h with 100 nM 4-HT prior to treatment with 2 μg ml^-1^ Tm for the indicated time. Lysates were fractionated by SDS-PAGE and analysed by immunoblotting with the indicated antibodies. Results in (A) and (B) are representative of 3 independent experiments. **(C)** NIH3T3 ΔCRAF:ER (left panel) or CCL39 ΔCRAF:ER (right panel) cells were pre-treated for 1 h with 100 nM 4-HT before addition of 2 μg ml^-1^ Tm for 48 h. Cells were then fixed, stained with propidium iodide and the proportion of cells with sub-G1 DNA was measured by flow cytometry. Results are the means ± S.D. of 3 independent experiments each performed in technical triplicate. Statistics represent the results of Student’s unpaired *t*-tests; **, p < 0.01; ***, p < 0.001. **(D)** COLO205 cells were treated for 24 h with 1 μM Selumetinib (Sel) in addition to DMSO, 30 nM Tg or 0.5 μg ml^-1^ Tm. Whole cell lysates were fractionated by SDS-PAGE and analysed by western blotting with the indicated antibodies. Results are representative of at least 3 independent experiments. **(E)** COLO205 cells were treated for 48 h as in (D). Cells were fixed, stained with propidium iodide and cell cycle distribution was determined by flow cytometry. Results are the means ± S.D. of an experiment performed in technical triplicate and representative of 3 independent experiments.

We investigated ER stress using two common ER stressors: thapsigargin (Tg), a sarco/endoplasmic reticulum Ca^2+^ ATPase (SERCA) inhibitor, depletes ER Ca^2+^, thereby undermining the protein folding capacity of the ER, whereas tunicamycin (Tm) inhibits N-linked glycosylation, promoting the accumulation of misfolded proteins within the ER lumen. Treatment of NIH3T3 fibroblasts with Tg or Tm resulted in a rapid expression of CHOP followed by the delayed expression of BiP and IRE1; these are characteristic markers of ER stress and UPR signalling (Fig 1A). Similar results were seen in several different fibroblast cell lines including iMEFs and CCL39 cells (see below) and in COLO205 colorectal cancer cells (S1A & S1B Fig). Both Tm and Tg promoted cell death in NIH3T3 cells as judged by the appearance of cleaved PARP (Fig 1B & S2A Fig) and by the appearance of cells with sub-G1 DNA (Fig 1C & S2B Fig); similar results were observed in CCL39 fibroblasts (Fig 1C & S2B Fig).

In the course of our analysis we noted that both Tg and Tm caused a progressive inactivation of the extracellular signal-regulated kinases, ERK1/2, as judged by the reduction in phosphorylated ERK1/2 (p-ERK1/2). This was observed in NIH3T3 fibroblasts (Fig 1A), CCL39 fibroblasts and immortalised mouse embryo fibroblasts (iMEFs). Prompted by this we examined the consequences of restoring ERK1/2 signalling using ΔCRAF:ER, a conditional mutant of CRAF that is activated by 4-hydroxytamoxifen (4HT) and drives MEK1/2-ERK1/2 signalling [28]. Treatment of NIH3T3 ΔCRAF:ER cells with 4HT prevented the Tm-induced loss of p-ERK1/2 and PARP cleavage (Fig 1B) and provided substantial protection against Tm-induced cell death (Fig 1C). Activation of ΔCRAF:ER did not prevent the Tm-induced expression of CHOP (Fig 1B) indicating that ERK1/2 signalling did not reduce ER stress or inhibit UPR signalling. Similar results were obtained in CCL39 ΔCRAF:ER cells [29] (Fig 1C) and when Tg replaced Tm as the ER stressor (S2A & S2B Fig). The protective effect of 4HT treatment was reversed by addition of selumetinib (AZD6244/ARRY-142886) [30] an allosteric MEK1/2 inhibitor (S3A & S3B Fig) or the selective ERK1/2 inhibitor SCH772984 [31] (S3C & S3D Fig). Furthermore, either selumetinib or SCH772984 increased Tg- and Tm-induced cell death in NIH3T3 cells (S3B & S3D Fig) suggesting that ERK1/2 signalling normally limits ER stress-induced cell death.

In contrast to these fibroblast cell lines, COLO205 cells maintained p-ERK1/2 levels following either Tg or Tm treatment (Fig 1D) and exhibited only a modest increase in cell death in response to these treatments (Fig 1E). COLO205 cells possess an activating BRAF^V600E^ mutation and exhibit constitutive ERK1/2 signalling which could drive innate resistance to ER stress. Indeed, whilst selumetinib caused little or no cell death when added to COLO205 cells alone [27, 32] it inhibited constitutive ERK1/2 signalling (Fig 1D) and combined with Tg or Tm to cause a striking increase in cell death (Fig 1E). Selumetinib also enhanced ER stress-induced cell death in the BRAF^V600E^ mutant colorectal cancer cell line, HT29 (S4 Fig). This effect was smaller than that seen in COLO205 cells and probably reflects the presence in HT29 cells of an activating p110α^P449T^ mutation in *PIK3CA* that may contribute MEK1/2-ERK1/2 independent survival signals; notably COLO205 cells exhibit wild type *PIK3CA*. Together with the experiments using ΔCRAF:ER these results demonstrated in multiple cell lines that ER stress-induced cell death is limited by the magnitude of ERK1/2 signalling and that ERK1/2 acts downstream or independently of canonical UPR signalling to protect cells.

### ER stress drives caspase-dependent cell death that is dependent upon BAX and BAK

**Fig 2:**
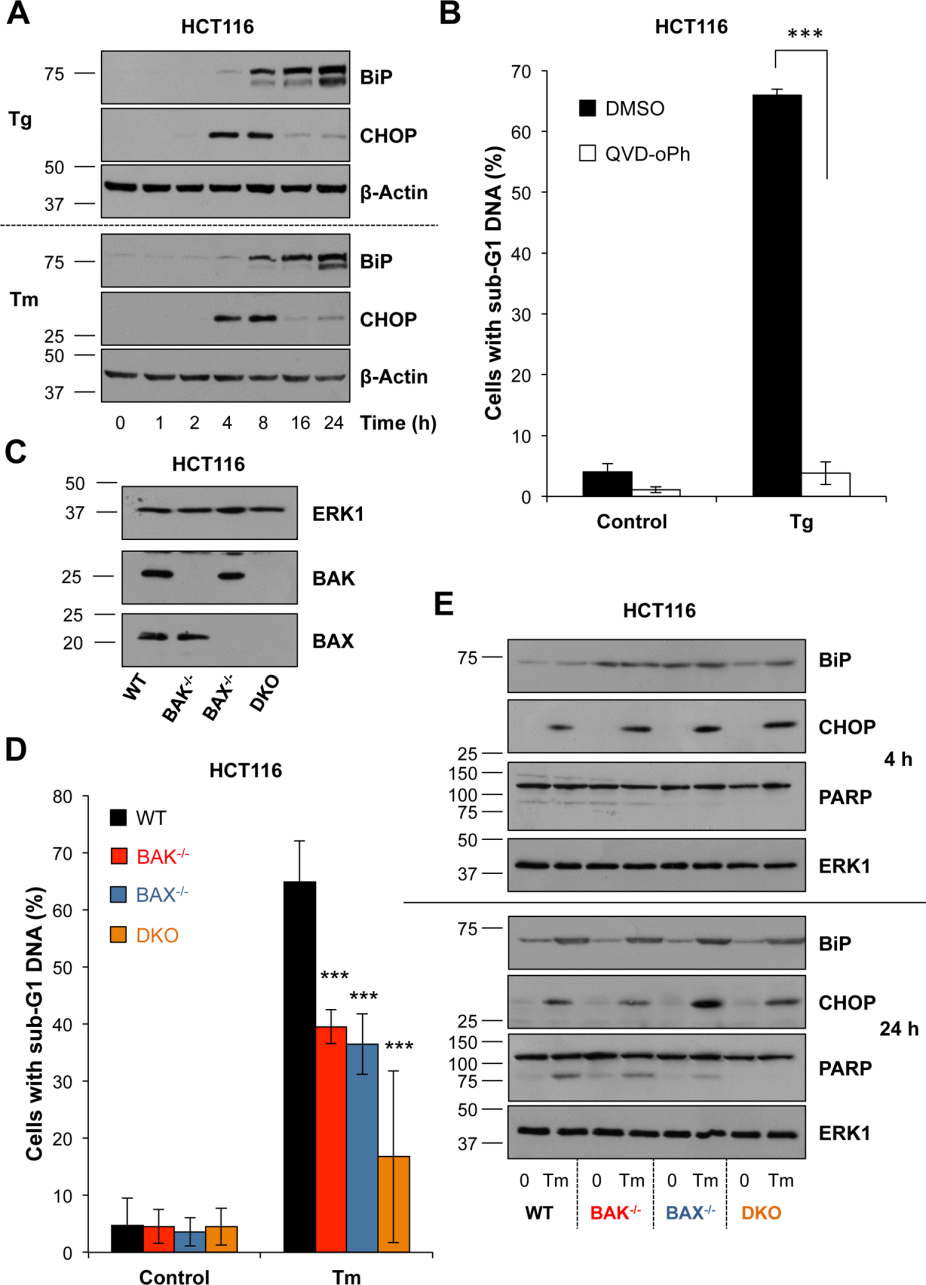
ER stress induces BAK/BAX-dependent, apoptotic cell death. **(A)** HCT116 cells were treated with 100 nM Tg (top panel) or 2 μg ml^-1^ Tm (bottom panel) for the indicated time, whole cell lysates were fractionated by SDS-PAGE and analysed by immunoblotting with the indicated antibodies. Results are representative of at least 3 independent experiments. **(B)** HCT116 cells were treated with 100 nM Tg in the presence or absence of 10 μM QVD-oPh for 48 h, fixed, stained with propidium iodide and the proportion of cells with sub-G1 DNA was measured by flow cytometry. Results are the means ± S.D. of 3 experiments each performed in technical triplicate. Student’s unpaired *t*-test results are indicated as follows; ***, p < 0.001. **(C)** Whole cell lysates of HCT116, HCT116 BAK^-/-^, HCT116 BAX^-/-^ or HCT116 BAK^-/-^, BAX^-/-^ (DKO) cells were separated by SDS-PAGE and immunoblotted with the indicated antibodies to confirm their genotype. **(D)** HCT116, HCT116 BAK^-/-^, HCT116 BAX^-/-^ or HCT116 BAK^-/-^, BAX^-/-^ (DKO) cells were treated with 2 μg ml^-1^ Tm for 48 h, fixed, stained with propidium iodide and the proportion of cells with sub-G1 DNA was measured by flow cytometry. Results are the means ± S.D. of 3 independent experiments each performed in technical triplicate. Statistics represent the results of two-way ANOVA and Bonferroni post-tests comparing each genotype to WT; ***, p < 0.001. **(E)** HCT116, HCT116 BAK^-/-^, HCT116 BAX^-/-^ or HCT116 BAK^-/-^, BAX^-/-^ (DKO) cells were treated with 2 μg ml^-1^ Tm for 4 h (top panel) or 24 h (bottom panel). Whole cell lysates were separated by SDS-PAGE and analysed by immunoblotting with the indicated antibodies. Results are representative of three independent experiments.

To understand how ERK1/2 signalling protected against ER stress we first defined the cell death response in detail using pharmacological and genetic interventions. Treatment of HCT116 cells with Tg or Tm resulted in the same coordinate changes in expression of CHOP and BiP (Fig 2A) and caused a striking increase in apoptosis as defined by the protective effect of the pan-caspase inhibitor QVD-oPh (Fig 2B). It has been proposed that ER stress-induced apoptosis proceeds by the cell intrinsic, mitochondrial pathway that is regulated by the BCL2 protein family [14, 21] but other studies have implicated death receptor signalling via DR5 [17, 24]. Using isogenic clones of HCT116 cells lacking either the BAX or BAK executioner proteins (Fig 2C) we found that Tm-induced apoptosis was reduced by loss of BAX or BAK but was almost completely abolished in BAX/BAK double knockout (DKO) cells (Fig 2D). These isogenic clones exhibited normal expression of CHOP and BiP in response to Tm (Fig 2E), so that defects in ER stress-induced death were not due to amelioration of ER stress or reduced UPR signalling. BAX/BAK DKO cells were also protected against Tg-induced cell death (S5A & S5B Fig). Together these results demonstrated that ER stress promoted caspase-dependent apoptotic cell death that was dependent upon BAX and BAK.

### ER stress fails to increase BIM expression but inhibits the expression of multiple pro-survival proteins

**Fig 3:**
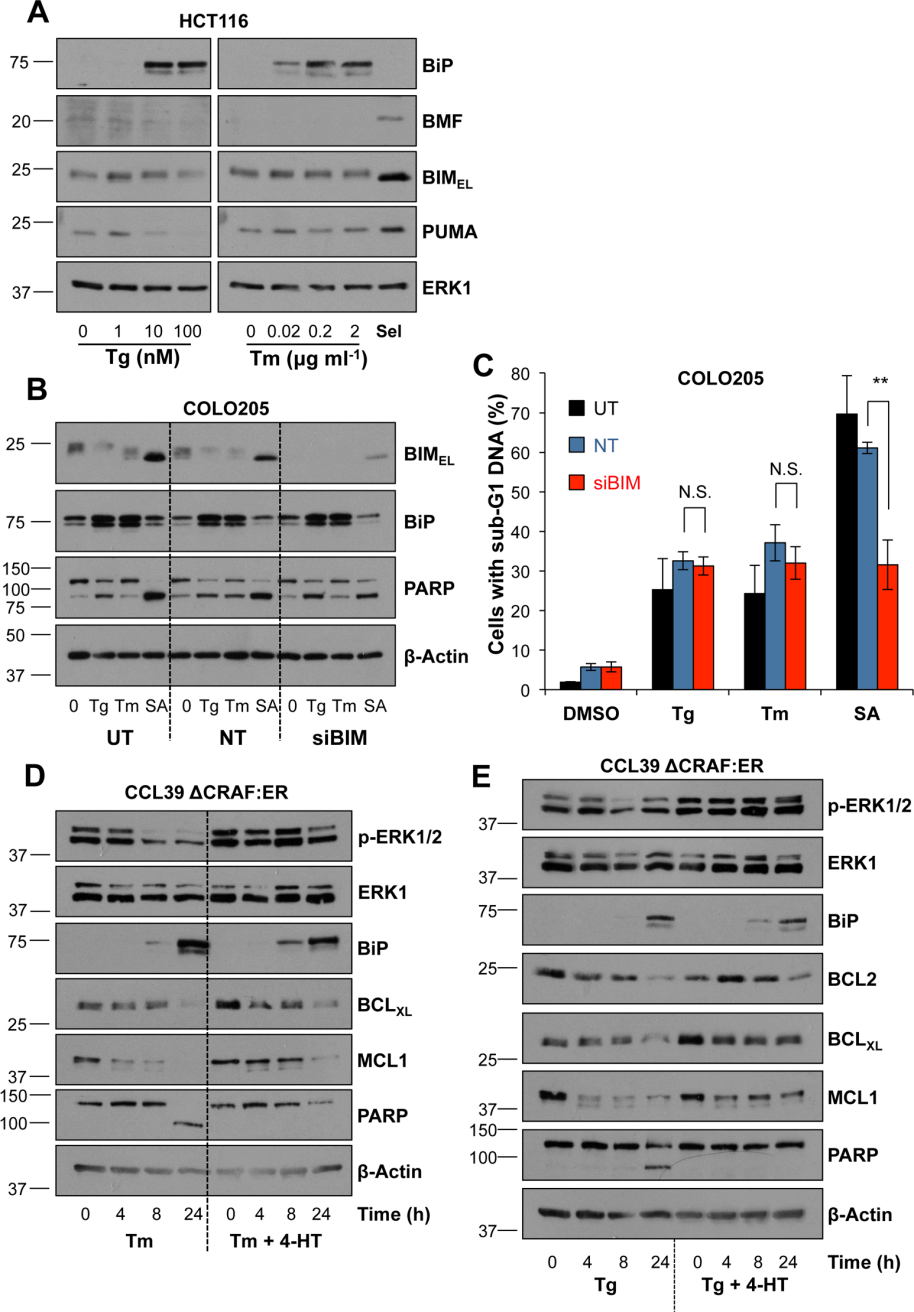
ER stress-induced apoptosis does not require BIM but is accompanied by loss of multiple pro-survival proteins. **(A)** HCT116 cells were treated with 1 μM Selumetinib (Sel) or the indicated concentration of Tg (left panel) or Tm (right panel) for 24 h, whole cell lysates were then separated by SDS-PAGE and analysed by immunoblotting with the indicated antibodies. Results are representative of 3 independent experiments. **(B)** COLO205 cells were transfected with non-targeting (NT) or BIM-specific (siBIM) siRNA or left untransfected (UT). At 48 h post-transfection cells were treated with 100 nM Tg, 2 μg ml^-1^ Tm or with 1 μM Selumetinib + 200nM ABT-263 (SA) for a further 24 h. Whole cell lysates were fractionated by SDS-PAGE and analysed by immunoblotting with the indicated antibodies. Results are representative of at least 3 independent experiments. **(C)** COLO205 cells were transfected and treated for 48 h as described in (B), fixed, stained with propidium iodide and the proportion of cells with sub-G1 DNA was measured by flow cytometry. Results are the means ± S.D. of 3 independent experiments each performed in technical triplicate, and statistics represent the results of Student’s unpaired *t*-tests; N.S., not significant; **, p < 0.01. **(D & E)** CCL39 ΔCRAF:ER cells were pre-treated for 1 h with 100 nM 4-HT prior to the addition of 2 μg ml^-1^ Tm (D) or 100 nM Tg (E) for the indicated time. Whole cell lysates were fractionated by SDS-PAGE and were transferred to immunoblot before analysis with the indicated antibodies. Results are representative of 3 independent experiments.

ER stress is proposed to activate apoptosis by increasing expression of BIM, a pro-apoptotic ‘BH3 only protein’ (BOP) and part of the BCL2 protein family [22, 23]. Indeed, this was consistent with prior observations that ERK1/2 signalling represses BIM [25, 33] and protects against ER stress (Fig 1; S2 Fig). However, neither Tg nor Tm treatment increased the expression of BIM_EL_, or BMF and PUMA, two other BOPs that are repressed by ERK1/2 signalling (Fig 3A) [27]. In contrast, selumetinib treatment increased the expression of BIM_EL_, BMF and PUMA and promoted the de-phosphorylation of BIM_EL_ (Fig 3A) indicating that we could detect dynamic changes in these proteins under appropriate conditions. Whilst there were no changes in BIM abundance, BIM could be activated by post-translational mechanisms [33] and contribute to apoptosis so we used RNA interference to investigate if BIM was required for ER stress-induced apoptosis. Despite very strong knock down (Fig 3B), BIM siRNA had no effect on Tg- or Tm-induced death in COLO205 cells (Fig 3C). As a control, BIM siRNA inhibited cell death induced by the combination of selumetinib and the BH3-mimetic ABT-263 (SA) by up to 50% (Fig 3C) confirming previous observations [27] and demonstrating that our assays were appropriate to detect BIM-dependent apoptosis. BIM induction by ER stress is proposed to be mediated by CHOP so we generated a HEK293 cell line that exhibited tetracycline-dependent expression of Myc-tagged CHOP (S6A Fig). This construct was functional since it promoted the expression of endogenous CHOP [34] (S6A Fig); however, Myc-CHOP failed to increase BIM levels (S6B Fig), even when its expression was combined with ER stress which might activate co-factors or promote required post-translational modifications [22, 35, 36].

The cell-intrinsic pathway of apoptosis can be initiated by the expression/activation of BH3-only proteins or the repression/inactivation of pro-survival BCL2 proteins [15]. Indeed, we found that Tg and Tm both promoted a time- and dose-dependent reduction in the expression of multiple pro-survival proteins including BCL2, BCL_XL_ and MCL1, over the same dose range at which they induced BiP expression (Fig 3D & 3E; S7A Fig). The reduction in MCL1 and BCL2 expression was apparent within 4–8 hours of Tg or Tm treatment, prior to any evidence of caspase activation as judged by PARP cleavage (Fig 3D & 3E). However, at 24 hrs when PARP cleavage was apparent the inclusion of the pan-caspase inhibitor QVD-oPh reversed some of the reduction in MCL1, BCL_XL_ and BCL2 expression and this effect was more apparent after 48 hours (S7B Fig). These results suggest that the early loss of pro-survival proteins is largely independent of caspase activation but caspase-dependent cleavage may make a progressively greater contribution at later times as the cells are dying. Notably, activation of ΔCRAF:ER-MEK1/2-ERK1/2 delayed or reduced the loss of BCL_XL_ and MCL1 (Fig 3D & 3E), even at early time points prior to PARP cleavage, correlating with the increased survival effects of ERK1/2 signalling described above (Fig 1C; S2B Fig). Together, these results demonstrated that ER stress failed to increase expression of BIM (or BMF and PUMA) and that BIM was not required for ER stress-induced death. Rather, ER stress reduced the expression of multiple pro-survival BCL2 proteins, three of which (BCL2, BCL_XL_ and MCL1) are known to be regulated by ERK1/2-dependent survival signalling [37].

### ER stress causes the PERK-dependent inhibition of translation and loss of pro-survival BCL2 proteins

**Fig 4:**
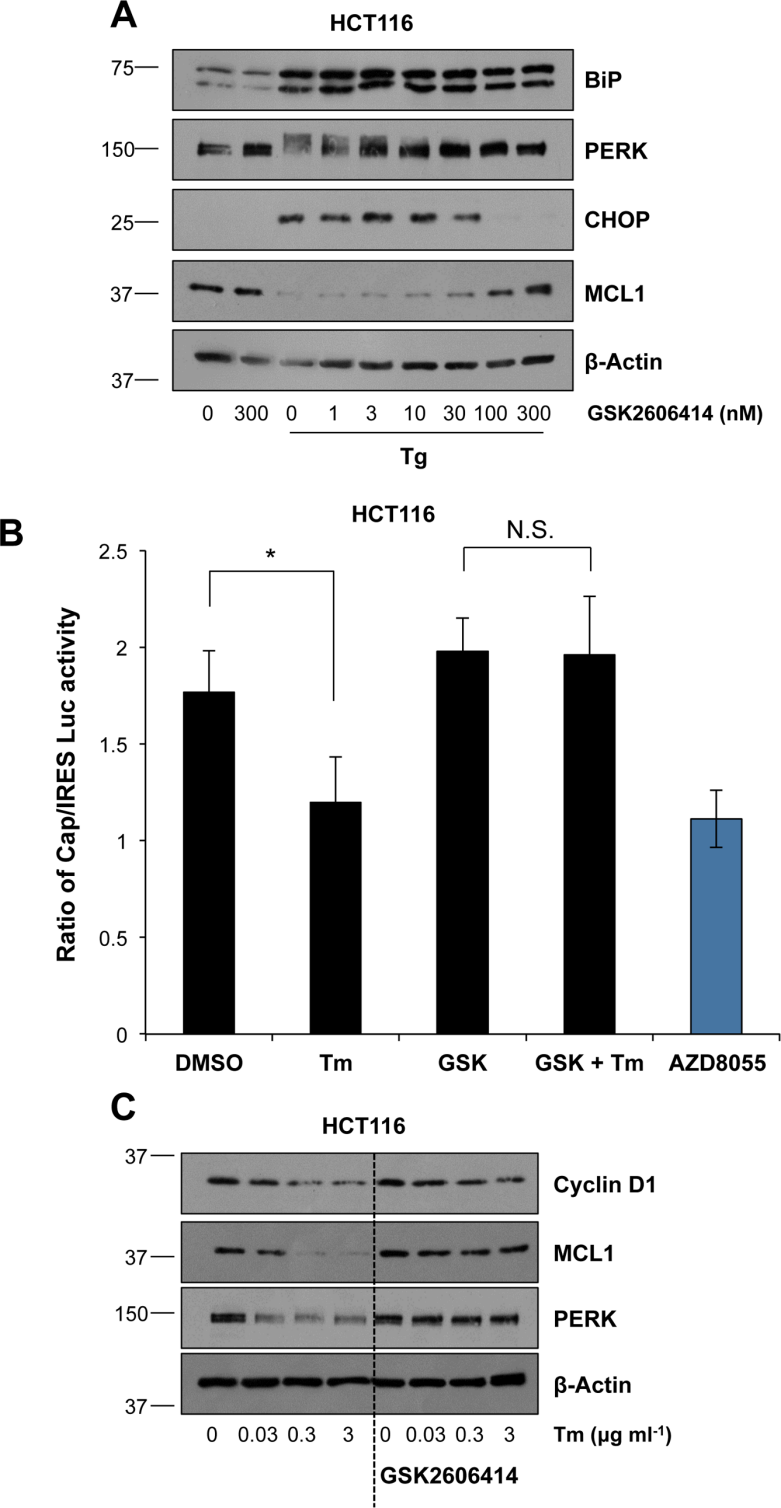
ER stress-induced inhibition of cap-dependent translation and loss of MCL1 is PERK-dependent. **(A)** HCT116 cells were pre-treated for 1 h with the indicated concentration of GSK2606414 before addition of 100 nM Tg for 6 h. Whole cell lysates were separated by SDS-PAGE and analysed by immunoblotting using the indicated antibodies. **(B)** HCT116 cells were transfected with a dual luciferase reporter construct for assay of CAP/IRES-dependent translation. 24 h post-transfection, cells were pre-treated for 1 h with 100 nM GSK2606414 (GSK) before addition of 2 μg ml^-1^ Tm or 1 μM AZD8055 for 24 h. Results shown are the mean ± S.D. luciferase activity within the whole cell lysates of one experiment performed in technical triplicate and are representative of three independent experiments. Statistics shown are the results of Student’s unpaired *t*-tests; N.S., not significant; *, p < 0.05. **(C)** HCT116 cells were pre-treated for 1 h with 100 nM GSK2606414 prior to the addition of the indicated concentration of Tm for 24 h. Whole cell lysates were fractionated by SDS-PAGE and analysed by immunoblotting using the indicated antibodies. Results in (A) and (C) are representative of 3 independent experiments.

ER stress inhibits global mRNA translation by PERK-dependent phosphorylation of eIF2α; this inhibits recruitment of the ribosome to the mRNA-bound pre-initiation complex. However, translation of the ATF4 transcription factor proceeds efficiently despite this due to a series of short upstream open reading frames within the *ATF4* 5′ UTR [7, 8] allowing ATF4 to drive the expression of target genes including CHOP. Following Tg or Tm treatment, PERK was autophosphorylated from 2 h onwards as determined by band-shift, although there were subtle differences in the effects of these different ER stressors after 8 h (S8A Fig), consistent with previous reports [38]. To assess whether the loss of MCL1 following ER stress was a result of this PERK-dependent pathway we used GSK2606414, a novel, potent and highly selective inhibitor of the PERK kinase domain [39]. The efficacy and selectivity of GSK2606414 was confirmed by showing that it inhibited Tg-induced PERK auto-phosphorylation and CHOP expression, but failed to inhibit BiP expression even at 100 nM, a dose that abolished CHOP expression (Fig 4A). This is consistent with BiP being a target of ATF6 signalling [40] and indicates that IRE1 and ATF6 signalling are sufficient to maintain induction of BiP in these cells. To assess the efficacy of GSK2606414 we employed a bicistronic dual Renilla–Firefly luciferase reporter construct (pRL-IRES-FL) which directs cap-dependent translation of the Renilla luciferase gene and cap-independent, polio IRES (polIRES)-mediated translation of the firefly luciferase gene [41,42]. Indeed, Tm treatment reduced the cap/IRES-dependent translation ratio, to a similar extent as that observed with the mTOR kinase inhibitor AZD8055, and this was completely reversed by the PERK inhibitor GSK2606414 (Fig 4B).

We then used GSK2606414 to investigate the role of PERK in the loss of MCL1. These experiments involved a 24 hour treatment with Tm during which PERK expression actually declined so that the hyper-phosphorylated forms of PERK were not readily visible, unlike with Tg treatment, where PERK levels recovered at 8 and 24 hours (S8A Fig); nonetheless GSK2606414 completely prevented this loss of PERK. Tm again caused a dose-dependent loss of expression of MCL1 and also cyclin D1, both encoded by mRNAs that undergo cap-dependent translation. GSK2606414 completely prevented the Tm-induced loss of cyclin D1 and MCL1 (Fig 4C) and also completely prevented the Tg-induced loss of MCL1 (Fig 4A) suggesting that this was due to PERK-dependent inhibition of translation. Thus ER stress acts through PERK to inhibit cap-dependent protein translation, including that of pro-survival proteins such as MCL1.

### Despite sustaining pro-survival protein levels, PERK inhibition enhances ER stress-induced death

**Fig 5:**
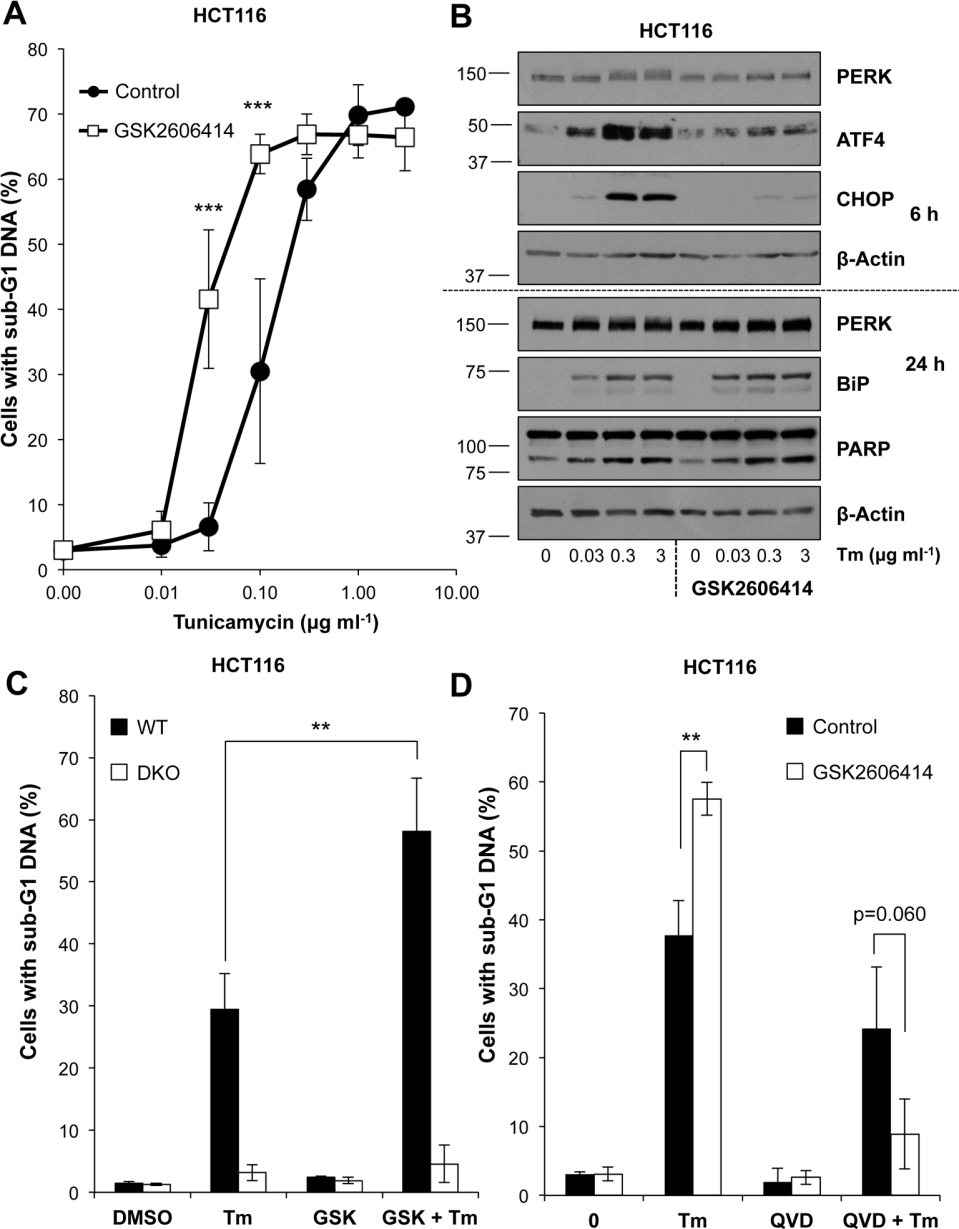
The PERK inhibitor GSK2606414 exacerbates ER stress-induced apoptosis. **(A)** HCT116 cells were pre-treated for 1 h with 100 nM GSK2606414 prior to addition of the indicated concentration of Tm for 48 h. Cells were fixed, stained with propidium iodide and the proportion of cells with sub-G1 DNA was measured by flow cytometry. Results shown are the means ± S.D. of 3 independent experiments each performed in technical triplicate. Statistics represent the results of two-way ANOVA and Bonferroni post-tests; ***, p < 0.001. **(B)** HCT116 cells were treated as in (A) for 6 h (top panel) or 24 h (bottom panel). Whole cell lysates were analysed by immunoblotting using the indicated antibodies following separation by SDS-PAGE. Results shown are representative of 3 independent experiments. **(C)** HCT116 and HCT116 BAK^-/-^, BAX^-/-^ (DKO) cells were pre-treated for 1 h with 100 nM GSK2606414 (GSK) before 48 h treatment with 0.1 μg ml^-1^ Tm. Cells were fixed, stained with propidium iodide and the proportion of cells with sub-G1 DNA was measured by flow cytometry. **(D)** HCT116 cells were pre-treated for 1 h with 100nM GSK2606414 prior to addition of 0.1 μg ml^-1^ Tm and 10 μM QVD-oPh (QVD), as indicated, for 48 h. Cells were fixed, stained with propidium iodide and the proportion of cells with sub-G1 DNA was measured by flow cytometry. Results shown in (C) and (D) are the means ± S.D. of 3 independent experiments performed in technical triplicate. Statistics represent the results of Student’s unpaired *t*-tests; **, p < 0.01.

**Fig 6:**
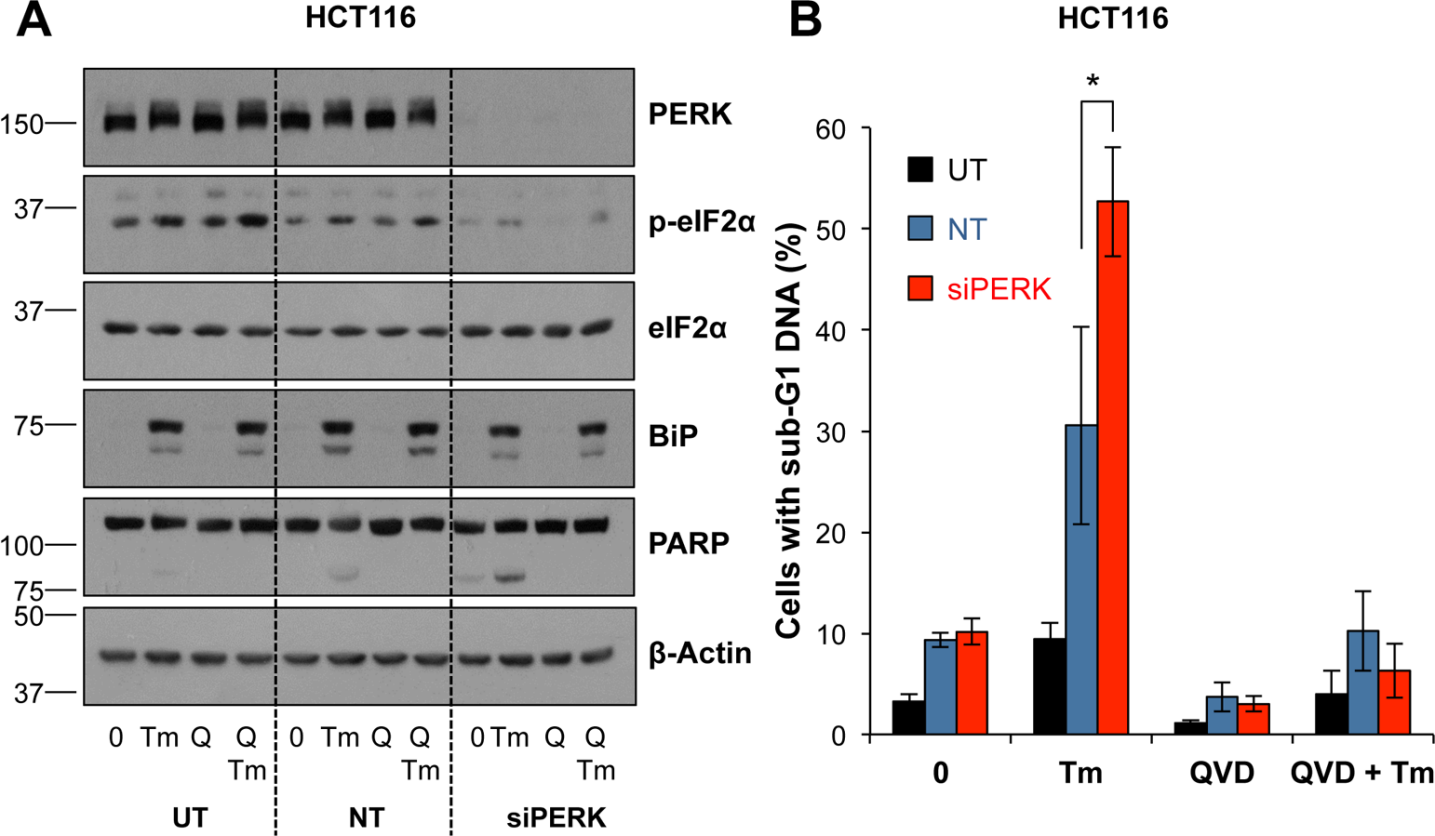
RNAi-mediated silencing of PERK exacerbates ER stress-induced apoptosis. **(A)** HCT116 cells were untransfected (UT) or transfected with non-targeting (NT) or PERK-targeting (siPERK) siRNA for 48 h prior to treatment with 0.1 μg ml^-1^ Tm and 10 μM QVD-oPh (Q) for 24 h. Whole cell lysates were fractionated by SDS-PAGE and analysed by immunoblotting using the indicated antibodies. Results shown are representative of three independent experiments. **(B)** HCT116 cells were transfected for 48 h as in (A) prior to treatment for 48 h with 0.1 μg ml^-1^ Tm and 10 μM QVD-oPh (QVD). Cells were fixed, stained with propidium iodide and the proportion of cells with sub-G1 DNA was measured by flow cytometry. Results are the combined means ± S.D. of three independent experiments performed in technical triplicate. Statistics represent the results of Student’s unpaired *t*-tests; *, p < 0.05.

**Fig 7:**
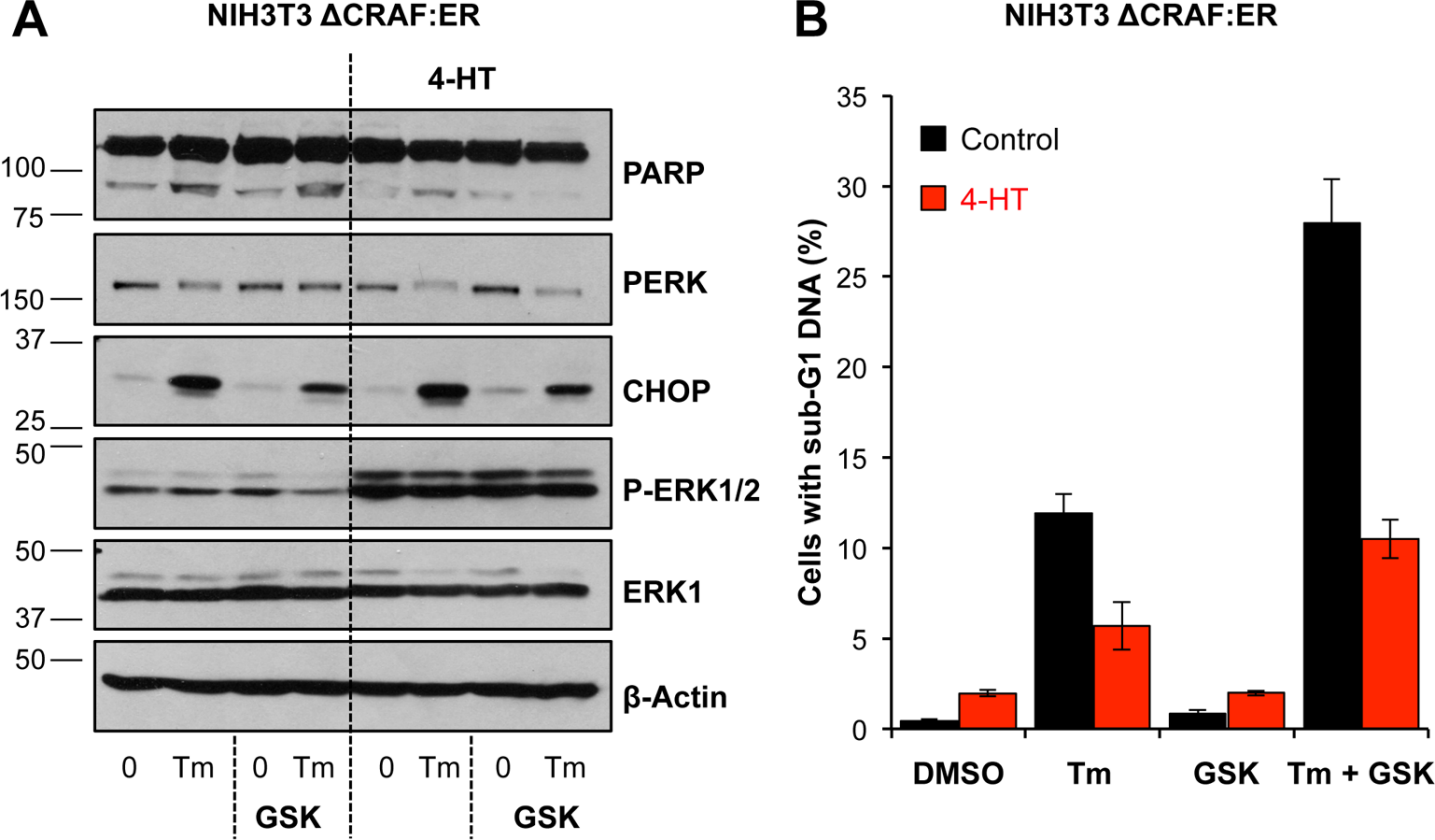
Activation of ERK1/2 protects against cell death arising from PERK inhibition. **(A)** NIH3T3 ΔCRAF:ER cells were pre-treated with 100 nM 4-HT for 1 h before addition of 100 nM GSK2606414 (GSK) and 0.1 μg ml^-1^ Tm alone or in combination as indicated for 24 h. Whole cell lysates were fractionated by SDS-PAGE and analysed by immunoblotting using the indicated antibodies. Results are representative of two independent experiments. **(B)** NIH3T3 ΔCRAF:ER cells were pre-treated with 100 nM 4-HT for 1 h before addition of 100 nM GSK2606414 (GSK) and 0.1 μg ml^-1^ Tm alone or in combination as indicated for 48 h. Cells were fixed and analysed by flow cytometry following propidium iodide staining. Results are means ± S.D. of three technical replicates from a single experiment and are representative of two independent experiments.

Although inhibition of PERK could sustain MCL1 levels we found that treatment with GSK2606414 actually promoted Tm-induced cell death (Fig 5A). Control blots confirmed that treatment with GSK2606414 inhibited PERK-dependent induction of ATF4 and CHOP, without affecting the later induction of BiP (Fig 5B). Similarly, GSK2606414 treatment enhanced Tg-induced cell death and this was inhibited by QVD-oPh (S8B Fig). The increase in Tm-induced cell death following PERK inhibition was still BAK/BAX-dependent (Fig 5C) and caspase-dependent (Fig 5D). To verify these results were due to PERK inhibition, PERK targeting siRNA was used and abolished PERK-dependent eIF2α phosphorylation without affecting Tm-induced BiP levels (Fig 6A). Consistent with the results using GSK2606414, PERK knockdown enhanced Tm-induced cell death and this was inhibited by addition of QVD-oPh (Fig 6B). Therefore despite maintenance of MCL1 levels, chemical or genetic PERK inhibition promoted caspase- and BAK/BAX-dependent cell death following ER stress demonstrating that PERK acts as a pro-survival pathway during ER stress. Finally, activation of ΔCRAF:ER-MEK1/2-ERK1/2 signalling not only protected against Tm, but was also able to protect against the enhanced cell death arising from Tm+GSK2606414 (Fig 7A & 7B). Thus PERK protects against Tm-induced death and PERK inhibition further enhances cell death through a pathway that is also inhibitable by ERK1/2 signalling, establishing that ERK1/2 activation can protect against cell death arising from PERK inhibition.

### ER stress induces ERK1/2-regulated, BAK/BAX-dependent apoptosis and a distinct ERK1/2- and BAK/BAX-independent cell death pathway

In previous studies, BAK/BAX DKO cells were not protected against prolonged ER stress [43]. Indeed, despite significant protection in short-term (48 hour) assays for apoptosis (Fig 2D; S5A Fig), BAK/BAX DKO cells were not protected against either Tm- or Tg-induced cell death in a long-term clonogenic assay (S9A Fig); similarly caspase inhibition did not promote long-term clonogenic survival of HCT116 cells against ER stress (S9B Fig). Finally, maintenance of ERK1/2 activity in NIH3T3 and CCL39 cells using ΔCRAF:ER prevented ER stress-induced apoptosis (Fig 1C; S2B Fig), but did not confer long-term cell survival (S9C Fig). Thus in addition to apoptosis, ER stress also induced a BAK/BAX-independent pathway of cell death, which was not inhibited by ERK1/2 activation.

**Fig 8:**
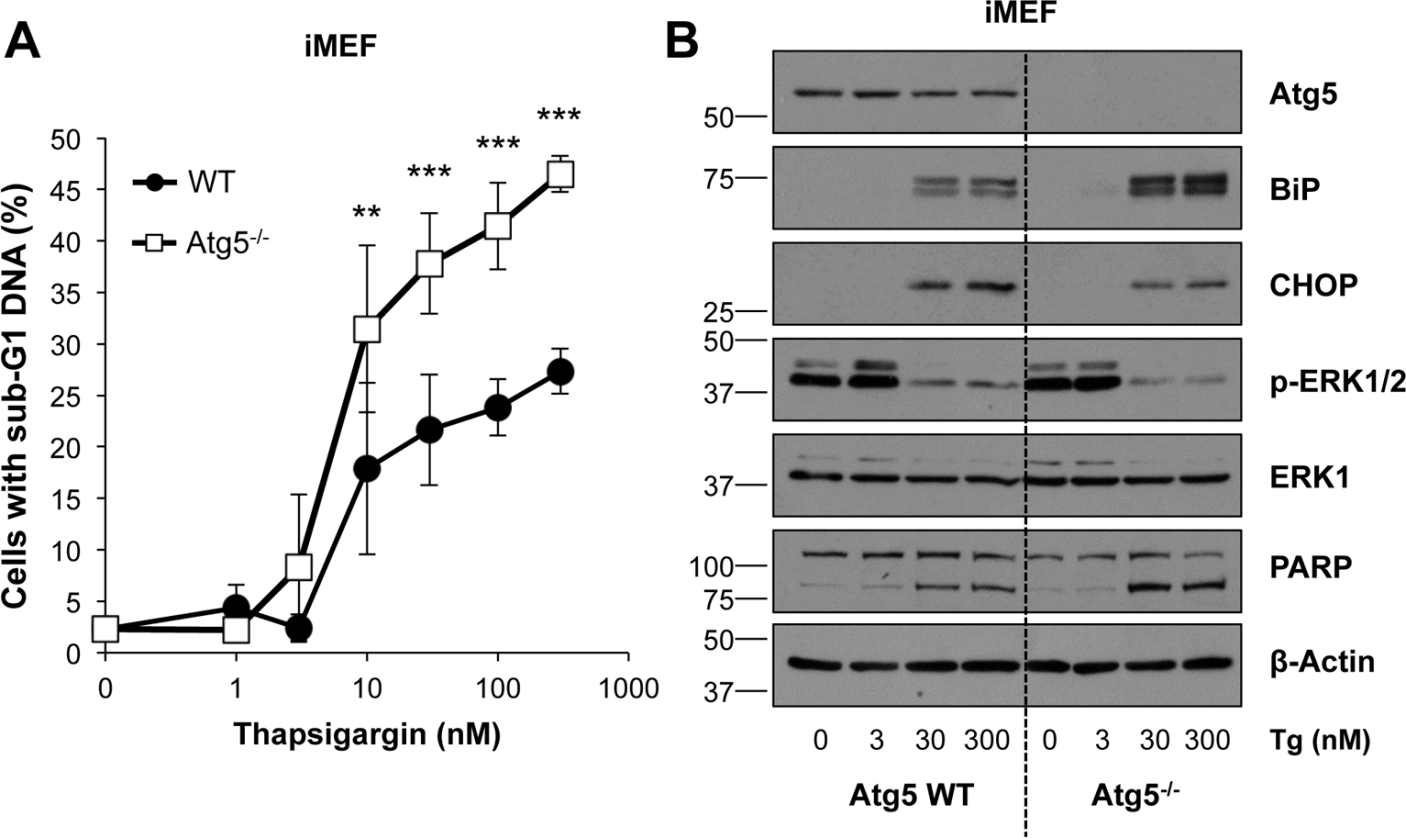
Atg5^-/-^ immortalised MEFs exhibit enhanced ER stress-induced cell death. **(A)** Atg5 WT and Atg5^-/-^ iMEFs were treated with the indicated concentration of Tg for 48 h. Cells were fixed and analysed by flow cytometry following propidium iodide staining. Results are the means ± S.D. of 3 independent experiments performed in technical triplicate. Statistics represent the results of two-way ANOVA followed by Bonferroni post-tests; **, p < 0.01; ***, p < 0.001. **(B)** Alternatively Atg5 WT and Atg5^-/-^ iMEFs were treated as in (A) for 24 h. Whole cell lysates were fractionated by SDS-PAGE prior to analysis by immunoblotting using the indicated antibodies. Results shown are representative of 3 independent experiments.

Autophagy has been proposed to promote both cell survival and cell death in response to ER stress [44], hence autophagy deficient (Atg5^-/-^) iMEFs were used to determine the role of autophagy following ER stress. Treatment with either ER stressors again decreased P-ERK1/2 and induced a greater proportion of cell death in Atg5^-/-^ iMEFs compared to WT iMEFs, though this effect was more pronounced for Tg treatment (Fig 8A; S10A Fig). Control blots demonstrated that Atg5^-/-^ iMEFs exhibited normal expression of the UPR markers, CHOP and BiP, following treatment with an ER stressor (Fig 8B; S10B Fig), so the increase in ER stress-induced cell death was not due to greater activation of UPR signalling. Therefore, induction of autophagy promoted cell survival and did not contribute to the alternative cell death pathway following ER stress.

## Discussion

Following ER stress, activation of the UPR leads to one of two outcomes; cell survival and adaptation, or cell death. There is a growing interest in engaging or selectively inhibiting one of these outcomes to manipulate the ER stress response as a therapeutic approach for certain disease indications [9, 45]. Here we investigated the role of the ERK1/2 signalling pathway in the cell death response induced by ER stress.

### ERK1/2 signalling limits ER stress-induced apoptosis

This study was prompted by the observation that Tg or Tm led to a progressive inactivation of ERK1/2 in several different fibroblast cell lines (Fig 1A and 1B; Fig 3D and 3E; Fig 8B; S10B Fig). This inactivation of ERK1/2 could be due to the induced expression of the dual specificity phosphatase (DUSP) enzymes that de-phosphorylate ERK1/2 [46]. However, ER stress did not inhibit ERK1/2 activation by ΔCRAF:ER (Fig 1B; S2A Fig); nor did it inhibit ERK1/2 in colorectal cancer cells with constitutively active BRAF^V600E^ (Fig 1D; S1 Fig). This suggests that the core RAF-MEK1/2-ERK1/2 pathway is largely unaffected and the loss of ERK1/2 activation following ER stress may be the result of decreased stimulation of the pathway upstream of RAF. This could arise through the expression of feedback regulators of the pathway such as sprouty (SPRY)/SPRY-related (SPRED) proteins that act at the level of receptors or RAS. Alternatively, many of the growth factors and cytokines that activate ERK1/2 signalling are trafficked through the ER and are sensitive to conditions within the ER lumen. Whilst there is a reduction in the luminal protein load in response to ER stress, there is also an increase in the secretion of pro-inflammatory cytokines [47–49]. So although ER stress does not induce a widespread loss of extracellular factors, it may promote a change in the secretion profile, perhaps favouring pro-inflammatory signalling (JNK, p38, NFκB) over ERK1/2 signalling. In addition, ER stress may also affect the synthesis, maturation and trafficking of the cell surface receptors for these growth factors and cytokines. For example, the ER luminal chaperone protein, BiP, which is induced in response to ER stress, inhibits the maturation of epidermal growth factor receptor (EGFR), promoting its retention within the ER and decreasing activation of downstream signalling pathways [50], including ERK1/2 signalling.

Whilst future studies should aim to define how ER stress impairs ERK1/2 signalling, our results indicate that ERK1/2 signalling is a key determinant of ER stress-induced apoptosis based on three key observations: (i) activation of ERK1/2 by ΔCRAF:ER protected cells from apoptosis; (ii) the MEK1/2 inhibitor selumetinib or the ERK1/2 inhibitor SCH772984 enhanced apoptosis induced by ER stress in fibroblasts and (iii) tumour cells with BRAF^V600E^ exhibited constitutive ERK1/2 signalling and MEK1/2-dependent resistance to ER stress. These results clearly demonstrate that ERK1/2 signalling normally limits the extent of ER stress-induced death in a variety of cell types and adds to growing evidence that this aspect of ERK1/2 survival signalling is co-opted by tumour cells; for example, MEK1/2 inhibition sensitises melanoma cells to ER stress-induced cell death [51]. Taken together with other studies [52,53], these results suggest that combining ERK1/2 pathway inhibitors with ER stressors, or ER stress mimetics that influence specific arms of the UPR, may have therapeutic potential in tumours with RAS, BRAF or MEK mutations [9]. By extension, ERK1/2 inhibition may influence responses to UPR inhibitors in other disease states characterised by ER stress [9].

### ER stress-induced apoptosis requires BAX/BAK but not BIM

Although ER stress has recently been proposed to induce apoptosis via activation of the DR5 death receptor [17, 24], it is unclear how this fits with prior reports of ER stress inducing BAK/BAX-dependent cell death [43] via increased BIM expression [22, 23]. Since BIM is a well-known target of ERK1/2 signalling [25,33,37] we anticipated that this would account for the cytoprotective effects of ERK1/2 during ER stress. However, whilst we found that ER stress-induced cell death was BAK/BAX-dependent (Fig 2D & S5 Fig), confirming dependence upon the intrinsic apoptotic pathway, we found no evidence for the involvement of BIM (Fig 3); ER stress did not increase BIM expression and BIM was not required for ER stress-induced apoptosis. We also examined BMF and PUMA, additional BOPs that are ERK1/2 targets [27], but again found no evidence that they were regulated by ER stress. Thus, engagement of BOPs is not a universal response to ER stress but rather depends on the cell type or perhaps the nature of the ER stressor [22, 54].

In addition to repressing BOPs, ERK1/2 signalling can promote survival by increasing the abundance of pro-survival BCL2 proteins [55]; for example, ERK1/2 signalling increases the transcription of MCL1 and BCL_XL_ and stabilizes the MCL1 protein [36,56–59]. We found that ER stress reduced the expression of multiple pro-survival BCL2 proteins including MCL1, BCL2 and BCL_XL_ and this was apparent at early time points prior to caspase activation (Fig 3D & 3E). In the case of MCL1 this was due to the PERK-dependent shut down of cap-dependent translation providing a tangible link to ER stress and the UPR; a link that we failed to detect for BIM, BMF or PUMA. Re-activation of ERK1/2 using ΔCRAF:ER, which prevents ER stress-induced apoptosis, was also able to sustain expression of MCL1, BCL2 and BCL_XL_ (Fig 3D & 3E). Indeed, ERK-dependent up-regulation of MCL1 has been proposed to account for the resistance of some melanoma cells to ER stress [60]. Taken together these results suggest that whilst ER stress-induced apoptosis is BAX/BAK-dependent it can proceed through the loss of pro-survival BCL2 proteins rather than the induction of pro-apoptotic BOPs such as BIM, BMF or PUMA.

### PERK inhibition enhances ER stress-induced cell death

The loss of MCL1 expression following ER stress was due to the PERK-dependent inhibition of cap-dependent translation (Fig 4); indeed, MCL1 is encoded by a cap-dependent transcript [61]. However, whilst chemical or genetic inhibition of PERK maintained the levels of MCL1, this did not protect against ER stress. Rather, chemical inhibition or genetic knockdown of PERK promoted BAX/BAK-dependent apoptosis (Figs 5 & 6) demonstrating that PERK normally functions to promote survival in the face of ER stress. It is known that inhibition of translation is critical in reducing the ER protein load following ER stress [62]; indeed, PERK^-/-^ cells are more sensitive to ER stress than WT cells [5, 62] presumably because PERK inhibition during ER stress sustains high levels of protein synthesis, thereby further exacerbating ER stress. ER stress in PERK^-/-^ iMEFs has been proposed to promote oxidative stress and expression of the BH3-only protein NOXA to drive apoptosis [63]. In the context of our study this is an attractive hypothesis since NOXA is the only selective MCL1 antagonist within the BOP division of BCL2 proteins. A recent study proposed that PERK signalling represses the caspase inhibitor XIAP [64], which in combination with the loss of pro-survival proteins shown here may sensitise the cell to apoptosis, but it is unclear how this would relate to ERK1/2-dependent protection against ER stress. However, whilst inhibition of apoptosis (loss of BAX/BAK or caspase inhibition) protected cells against combined ER stress and PERK inhibition in short-term assays (Fig 5) it could not provide protection in long-term clonogenic assays (S9A & S9B Fig). Similarly, whilst activation of ERK1/2 signalling by ΔCRAF:ER* prevented apoptosis arising from ER stress (Fig 1) or ER stress and PERK inhibition (Fig 7), it could not confer long-term protection, implying an alternative cell death pathway (S9C Fig).

Analysis of Atg5^-/-^ fibroblasts argued against autophagy contributing to cell death; rather, autophagy-deficient Atg5^-/-^ fibroblasts exhibited enhanced cell death suggesting that autophagy normally protects against ER stress, presumably by providing an alternative pathway for the removal of misfolded or damaged proteins. ER stress has been proposed to induce ATP depletion and necrotic cell death in cells deficient in BAX and BAK [65]; indeed, cells can switch between apoptosis and necroptosis when one or other cell death modality is inhibited [66]. Our own results are consistent with these reports and suggest that ER stress induces cell death by at least two biochemically and genetically distinct pathways: a classical BAX/BAK-dependent apoptotic response that can be inhibited by ERK1/2 signalling and an alternative, ERK1/2- and BAX/BAK-independent necrotic response. In addition, chronic ER stress may drive an irreversible cell cycle arrest and senescence and this may account for the strong reduction in clonogenic growth in BAX/BAK DKO cells. Indeed, persistent ERK1/2 signalling can drive senescence in primary cells [67] and cell cycle arrest in immortalised cells [28] this may explain why ERK1/2 activation by ΔCRAF:ER could not rescue the decline in clonogenicity following ER stress. Regardless, the finding that ER stress can switch between ERK1/2-regulated and ERK1/2-independent cell death pathways may be relevant to attempts to target ER stress signalling in certain pathologies.

## Supporting information

S1 Fig ER stress does not decrease P-ERK1/2 in COLO205 cells. **(A & B)** COLO205 cells were treated for the indicated time with 100 nM Tg (A) or 2 μg ml^-1^ Tm (B), and whole cell lysates were analysed by immunoblotting using the indicated antibodies following fractionation by SDS-PAGE. Results are representative of three independent experiments.
(TIF)

S2 Fig Activation of ΔCRAF:ER protects against Tg-induced cell death. (**A)** NIH3T3 ΔCRAF:ER cells were treated for 1 h with 100 nM 4-HT, prior to addition of 100 nM Tg for the indicated time. Whole cell lysates were fractionated by SDS-PAGE and analysed by immunoblotting using the indicated antibodies. Results shown are representative of three independent experiments. **(B)** NIH3T3 ΔCRAF:ER (left panel) or CCL39 ΔCRAF:ER (right panel) cells were pre-treated for 1 h with 100 nM 4-HT before addition of 100 nM Tg for 48 h. Cells were then fixed, stained with propidium iodide and cell cycle distribution was measured by flow cytometry. Results are the means ± S.D. from at least 3 independent experiments each performed in technical triplicate. Student’s unpaired *t*-test results are indicated as follows; ***, p < 0.001.
(TIF)

S3 Fig Protection against ER stress afforded by ΔCRAF:ER is dependent on MEK1/2 and ERK1/2. **(A)** NIH3T3 ΔCRAF:ER cells were pre-treated for 1 h with 100 nM 4-HT or 3 μM Selumetinib (Sel), prior to addition of DMSO, 100 nM Tg or 2 μg ml^-1^ Tm for 24 h. Whole cell lysates were analysed by immunoblotting using the indicated antibodies following fractionation by SDS-PAGE. Results shown are representative of two independent experiments. **(B)** NIH3T3 ΔCRAF:ER cells were pre-treated for 1 h with 100 nM 4-HT or 3 μM Selumetinib (Sel), prior to addition of DMSO, 100 nM Tg or 2 μg ml^-1^ Tm for 48 h. Cells were fixed, stained with propidium iodide and analysed by flow cytometry. Results shown are means ± S.D. of a single experiment performed in technical triplicate and representative of two independent experiments. Statistics represent the results of Student’s unpaired *t*-tests; **, p < 0.01; ***, p < 0.001. **(C)** NIH3T3 ΔCRAF:ER cells were pre-treated for 1 h with 100 nM 4-HT or 100 nM SCH772984 (SCH), prior to addition of DMSO, 100 nM Tg or 2 μg ml^-1^ Tm for 24 h. Whole cell lysates were analysed by immunoblotting using the indicated antibodies following fractionation by SDS-PAGE. Results shown are representative of two independent experiments. **(D)** NIH3T3 ΔCRAF:ER cells were pre-treated for 1 h with 100 nM 4-HT or 100 nM SCH772984 (SCH), prior to addition of DMSO, 100 nM Tg or 2 μg ml^-1^ Tm for 48 h. Cells were fixed, stained with propidium iodide and analysed by flow cytometry. Results shown are means ± S.D. of a single experiment performed in technical triplicate and representative of two independent experiments. Statistics represent the results of Student’s unpaired *t*-tests; **, p < 0.01; ***, p < 0.001.
(TIF)

S4 Fig MEK1/2-ERK1/2 signalling limits the extent of ER-stress induced cell death in HT29 cells. **(A)** HT29 cells were treated for 24 h with 1 μM Selumetinib (Sel) in addition to either DMSO or 30 nM Tg. Whole cell lysates were separated by SDS-PAGE and analysed by immunoblotting using the indicated antibodies. Results shown are representative of three independent experiments. **(B)** HT29 cells were treated as in (A) for 48 h, cells were fixed and stained with propidium iodide prior to analysis by flow cytometry. **(C)** HT29 cells were treated with 1 μM Selumetinib (Sel) and DMSO or 0.5 μg ml^-1^ Tm. Whole cell lysates were analysed by immunoblotting using the indicated antibodies following fractionation by SDS-PAGE. Results are representative of three independent experiments. **(D)** HT29 cells were treated for 48 h as described in (C), cells were fixed, stained with propidium iodide and analysed by flow cytometry. Results shown in (B) and (D) are means ± S.D. of a single experiment performed in technical triplicate and representative of three independent experiments. Statistics represent the results of Student’s unpaired *t*-tests; **, p < 0.01.
(TIF)

S5 Fig Tg-induced cell death in BAX/BAK dependent. **(A)** HCT116, HCT116 BAK^-/-^, HCT116 BAX^-/-^ or HCT116 BAK^-/-^, BAX^-/-^ (DKO) cells were treated with 100 nM Tg for 48 h. Cells were fixed and analysed by flow cytometry following propidium iodide staining. Results are means ± S.D. of three independent experiments performed in technical triplicate. Statistics represent the results of two-way ANOVA and Bonferroni post-tests comparing each genotype to WT; N.S., not significant; ***, p < 0.001. **(B)** HCT116, HCT116 BAK^-/-^, HCT116 BAX^-/-^ or HCT116 BAK^-/-^, BAX^-/-^ (DKO) cells were treated with 100 nM Tg for 4 h (top panel) or 24 h (bottom panel). Whole cell lysates were separated by SDS-PAGE and analysed by immunoblotting with the indicated antibodies. Results are representative of three independent experiments.
(TIF)

S6 Fig CHOP fails to induce BIM expression even when combined with ER stress. **(A)** HEK293 TetR cells stably transfected with pcDNA4/TO (EV) or pcDNA4/TO Myc-CHOP were treated for 4 h with 1 μg ml^-1^ tetracycline (Tet), 100 nM Tg or 2 μg ml^-1^ Tm. Whole cell lysates were analysed by immunoblotting following separation by SDS-PAGE. **(B)** Cell lines detailed in (A) were pre-treated for 2 h with 1 μg ml^-1^ tetracycline (Tet) followed by addition of 2 μg ml^-1^ Tm. Whole cell lysates were fractionated by SDS-PAGE and analysed by immunoblotting using the indicated antibodies. Results in (A) and (B) are from a single experiment, with comparable results detected in two inducible CHOP cell lines.
(TIF)

S7 Fig Loss of pro-survival BCL2 proteins upon ER stress and the effect of caspase inhibition. (A) COLO205 cells were treated with either Tg or Tm for 24 h. Whole cell lysates were fractionated by SDS-PAGE and analysed by immunoblotting using the indicated antibodies. Results shown are representative of three independent experiments. (B) HCT116 cells were pre-treated for 1 h with vehicle (C) or 10 μM QVD-oPH (Q) followed by 100 nM Tg or 2 μg ml^-1^ Tm for 24 or 48 h. Whole cell lysates were fractionated by SDS-PAGE and analysed by immunoblotting using the indicated antibodies. Results are representative of three independent experiments.
(TIF)

S8 Fig PERK inhibition enhances Tg-induced apoptosis. **(A)** HCT116 cells were treated with 100 nM Tg or 2 μg ml^-1^ Tm for the indicated times. Whole cell lysates were fractionated by SDS-PAGE and analysed by immunoblotting using the indicated antibodies. Results are from a single experiment. **(B)** HCT116 cells were pre-treated for 1 h with 100 nM GSK2606414 prior to addition of 10 nM Tg and 10 μM QVD-oPh (QVD) for 48 h. Cells were fixed and analysed by flow cytometry following propidium iodide staining. Results shown are the means ± S.D. for a single experiment, representative of two independent experiments performed in technical triplicate. Statistics represent the results of Student’s unpaired *t*-tests; **, p < 0.01.
(TIF)

S9 Fig Inhibition of apoptosis or activation of ΔCRAF:ER fails to confer long term protection against ER stress. **(A)** HCT116 or HCT116 BAK^-/-^, BAX^-/-^ (DKO) cells were treated for 72 h with 100 nM Tg (left panel) or 2 μg ml^-1^ Tm (right panel), treatment media was then removed and colonies were left to grow for 7 days. Cells were then fixed, stained with crystal violet and colonies with a diameter greater than approximately 0.2 mm were counted. Results shown are the combined means ± S.D. of three independent experiments performed in technical triplicate. **(B)** HCT116 cells were treated for 72 h with 10 μM QVD-oPh and either 100 nM Tg (left panel) or 2 μg ml^-1^ Tm (right panel) and colonies were analysed as in (A). Results shown are the means ± S.D. for a single experiment performed in technical triplicate. **(C)** NIH3T3 ΔCRAF:ER cells were pre-treated for 24 h with 100 nM 4-HT, prior to addition of 100 nM Tg (left panel) or 2 μg ml^-1^ Tm (right panel) for 24 h. Treatment media was removed and colonies were left to grow for 7 days before crystal violet absorbance was measured following staining. Results shown are from the combined means ± S.D. of five independent experiments performed in technical triplicate, displayed as the % staining compared to DMSO treated control and coefficient of variation. Student’s unpaired *t*-tests comparing colonies in the presence and absence of ER stressor in (A) and (C) are indicated as; *, p < 0.05; **, p < 0.01; ***, p < 0.001.
(TIF)

S10 Fig Tm-induced cell death in wild type and Atg5^-/-^ iMEFs. **(A)** Atg5 WT and Atg5^-/-^ iMEFs were treated for 48 h with the indicated concentration of Tm. Cells were fixed and analysed by flow cytometry following propidium iodide staining. Results shown are the combined means ± S.D. of three independent experiments performed in technical triplicate. **(B)** Atg5 WT and Atg5^-/-^ iMEFs were treated with the indicated concentration of Tm for 24 h. Whole cell lysates were analysed by immunoblotting using the indicated antibodies after fractionation by SDS-PAGE. Results shown are representative of three independent experiments.
(TIF)
