# Supplementary figures and images for: ERK1/2 signalling protects against apoptosis following endoplasmic reticulum stress but cannot provide long-term protection against BAX/BAK-independent cell death
Source: PLoS One. 2017 Sep 20;12(9):e0184907. doi: 10.1371/journal.pone.0184907 (PMC5607168; doi:10.1371/journal.pone.0184907)

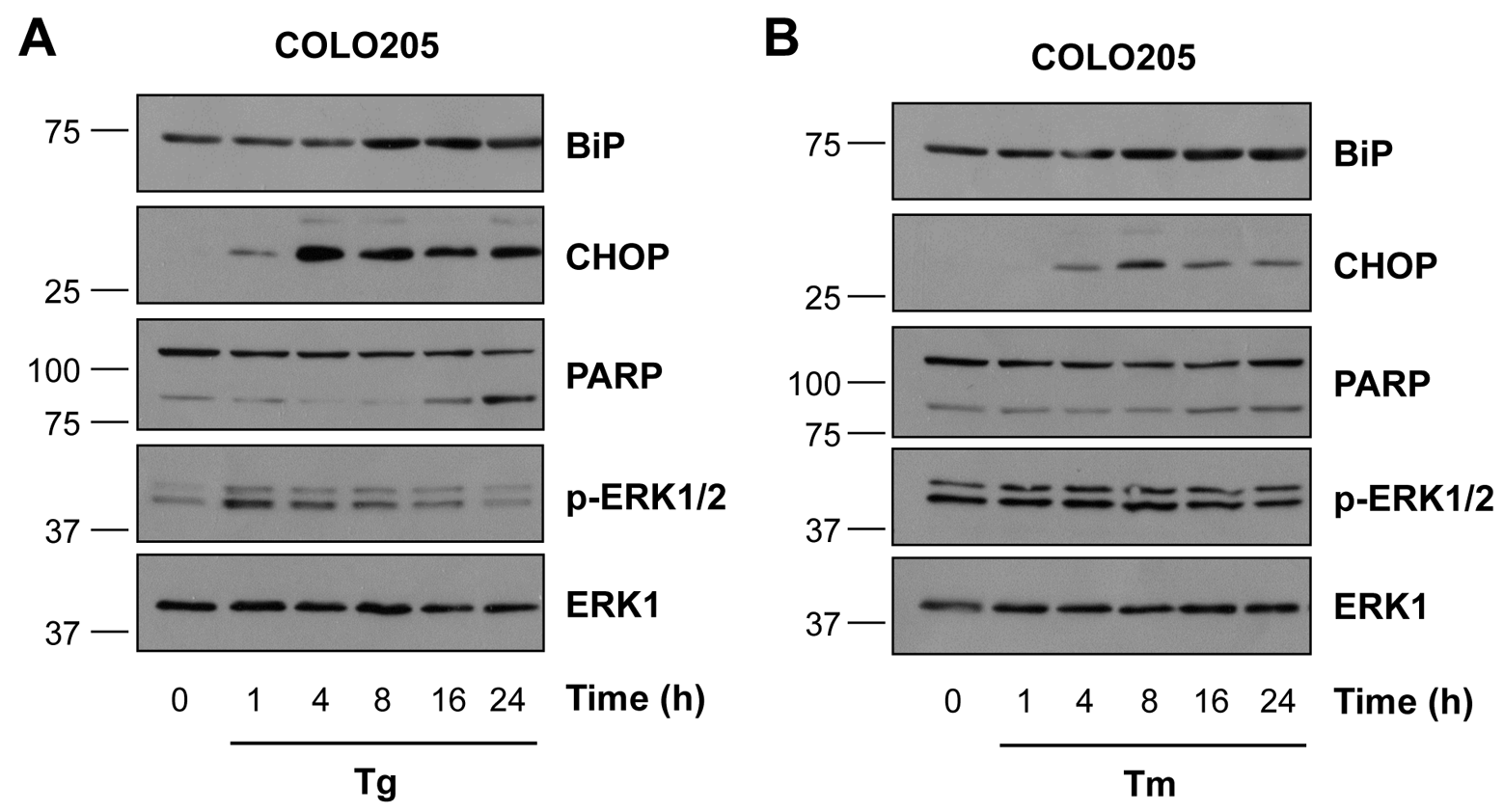

Supplement: S1 Fig — (A & B) COLO205 cells were treated for the indicated time with 100 nM Tg (A) or 2 μg ml-1 Tm (B), and whole cell lysates were analysed by immunoblotting using the indicated antibodies following fractionation by SDS-PAGE. Results are representative of three independent experiments. (TIF) [file pone.0184907.s001.tif]

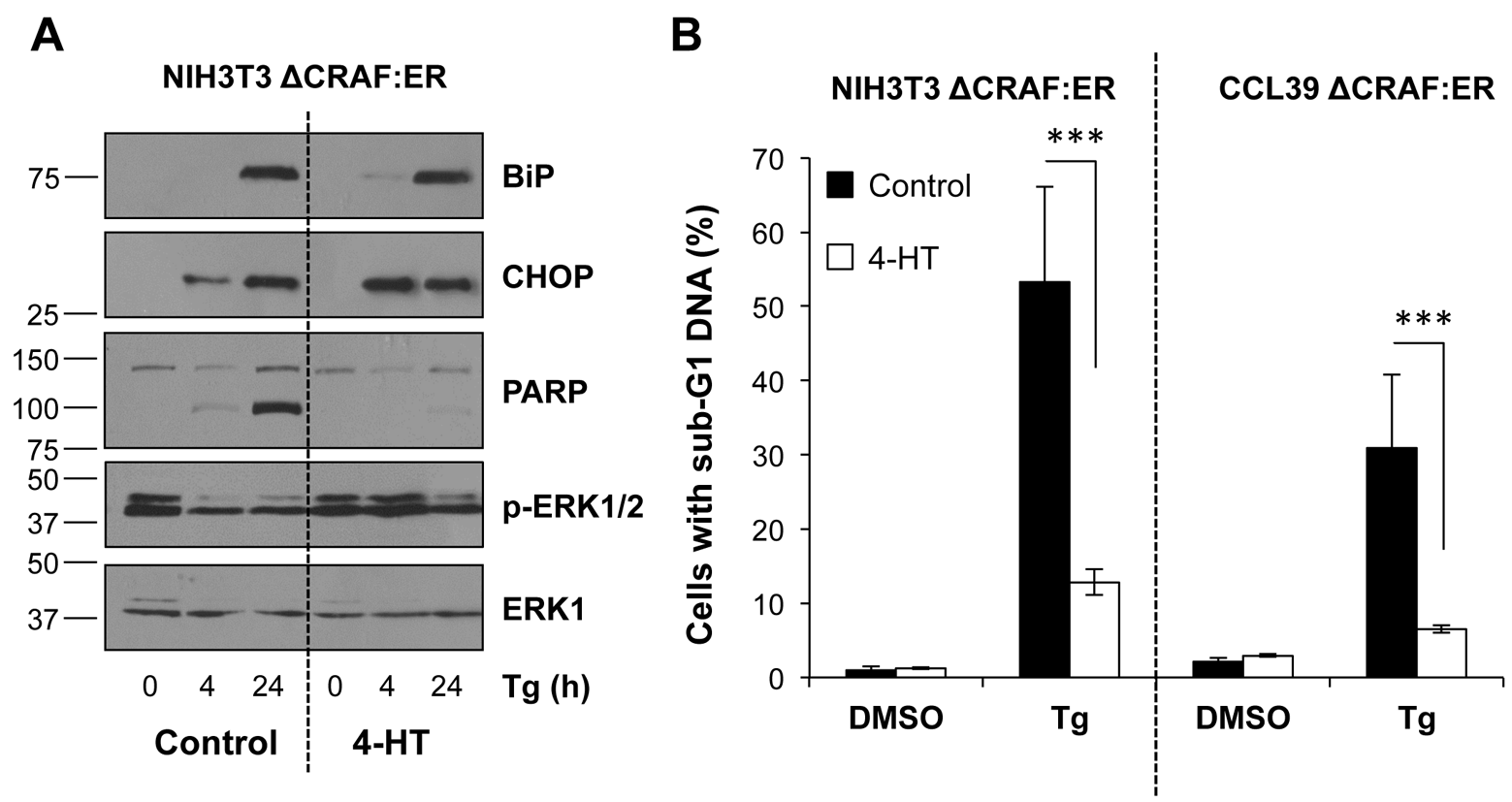

Supplement: S2 Fig — (A) NIH3T3 ΔCRAF:ER cells were treated for 1 h with 100 nM 4-HT, prior to addition of 100 nM Tg for the indicated time. Whole cell lysates were fractionated by SDS-PAGE and analysed by immunoblotting using the indicated antibodies. Results shown are representative of three independent experiments. (B) NIH3T3 ΔCRAF:ER (left panel) or CCL39 ΔCRAF:ER (right panel) cells were pre-treated for 1 h with 100 nM 4-HT before addition of 100 nM Tg for 48 h. Cells were then fixed, stained with propidium iodide and cell cycle distribution was measured by flow cytometry. Results are the means ± S.D. from at least 3 independent experiments each performed in technical triplicate. Student’s unpaired t-test results are indicated as follows; ***, p < 0.001. (TIF) [file pone.0184907.s002.tif]

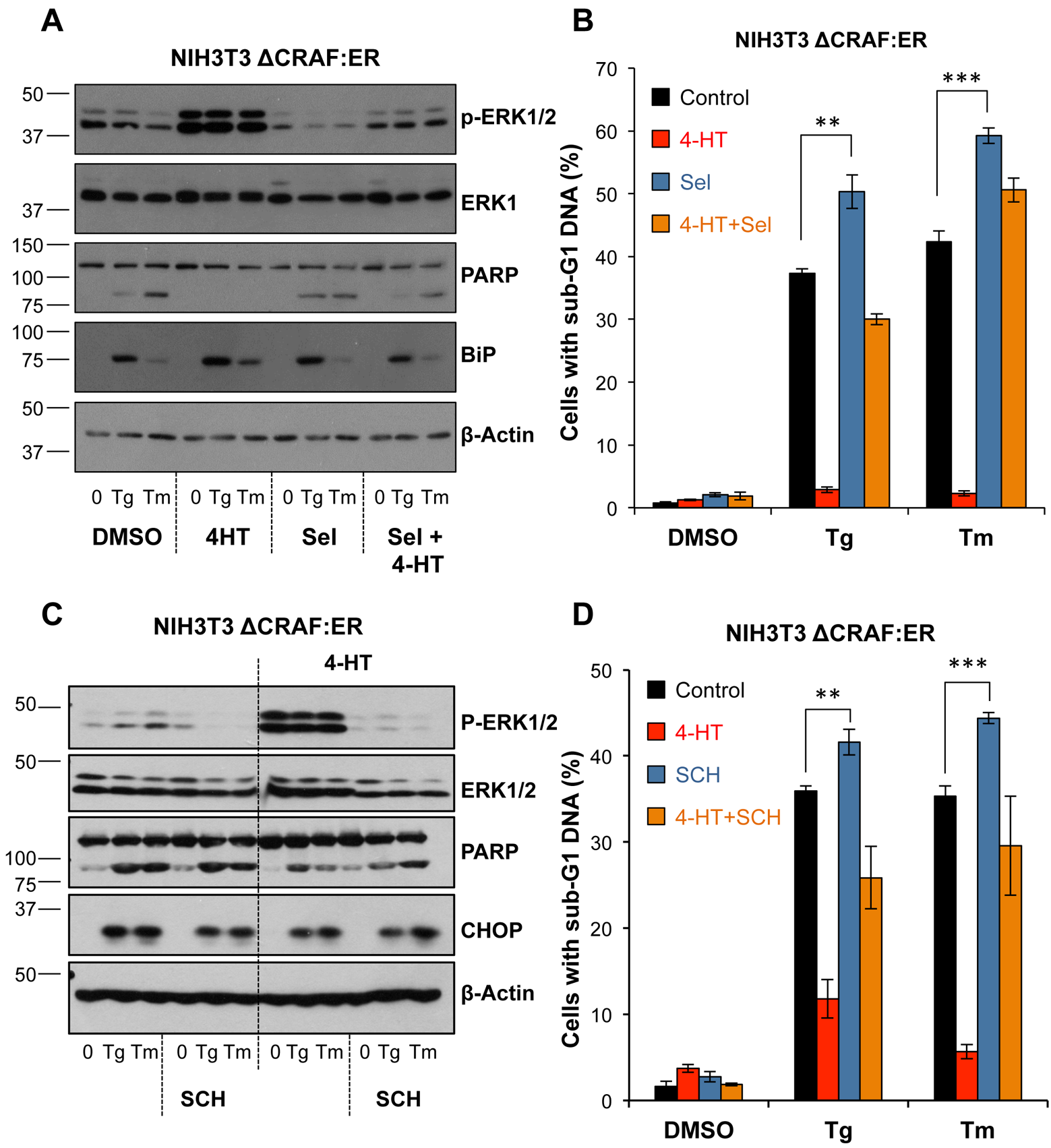

Supplement: S3 Fig — (A) NIH3T3 ΔCRAF:ER cells were pre-treated for 1 h with 100 nM 4-HT or 3 μM Selumetinib (Sel), prior to addition of DMSO, 100 nM Tg or 2 μg ml-1 Tm for 24 h. Whole cell lysates were analysed by immunoblotting using the indicated antibodies following fractionation by SDS-PAGE. Results shown are representative of two independent experiments. (B) NIH3T3 ΔCRAF:ER cells were pre-treated for 1 h with 100 nM 4-HT or 3 μM Selumetinib (Sel), prior to addition of DMSO, 100 nM Tg or 2 μg ml-1 Tm for 48 h. Cells were fixed, stained with propidium iodide and analysed by flow cytometry. Results shown are means ± S.D. of a single experiment performed in technical triplicate and representative of two independent experiments. Statistics represent the results of Student’s unpaired t-tests; **, p < 0.01; ***, p < 0.001. (C) NIH3T3 ΔCRAF:ER cells were pre-treated for 1 h with 100 nM 4-HT or 100 nM SCH772984 (SCH), prior to addition of DMSO, 100 nM Tg or 2 μg ml-1 Tm for 24 h. Whole cell lysates were analysed by immunoblotting using the indicated antibodies following fractionation by SDS-PAGE. Results shown are representative of two independent experiments. (D) NIH3T3 ΔCRAF:ER cells were pre-treated for 1 h with 100 nM 4-HT or 100 nM SCH772984 (SCH), prior to addition of DMSO, 100 nM Tg or 2 μg ml-1 Tm for 48 h. Cells were fixed, stained with propidium iodide and analysed by flow cytometry. Results shown are means ± S.D. of a single experiment performed in technical triplicate and representative of two independent experiments. Statistics represent the results of Student’s unpaired t-tests; **, p < 0.01; ***, p < 0.001. (TIF) [file pone.0184907.s003.tif]

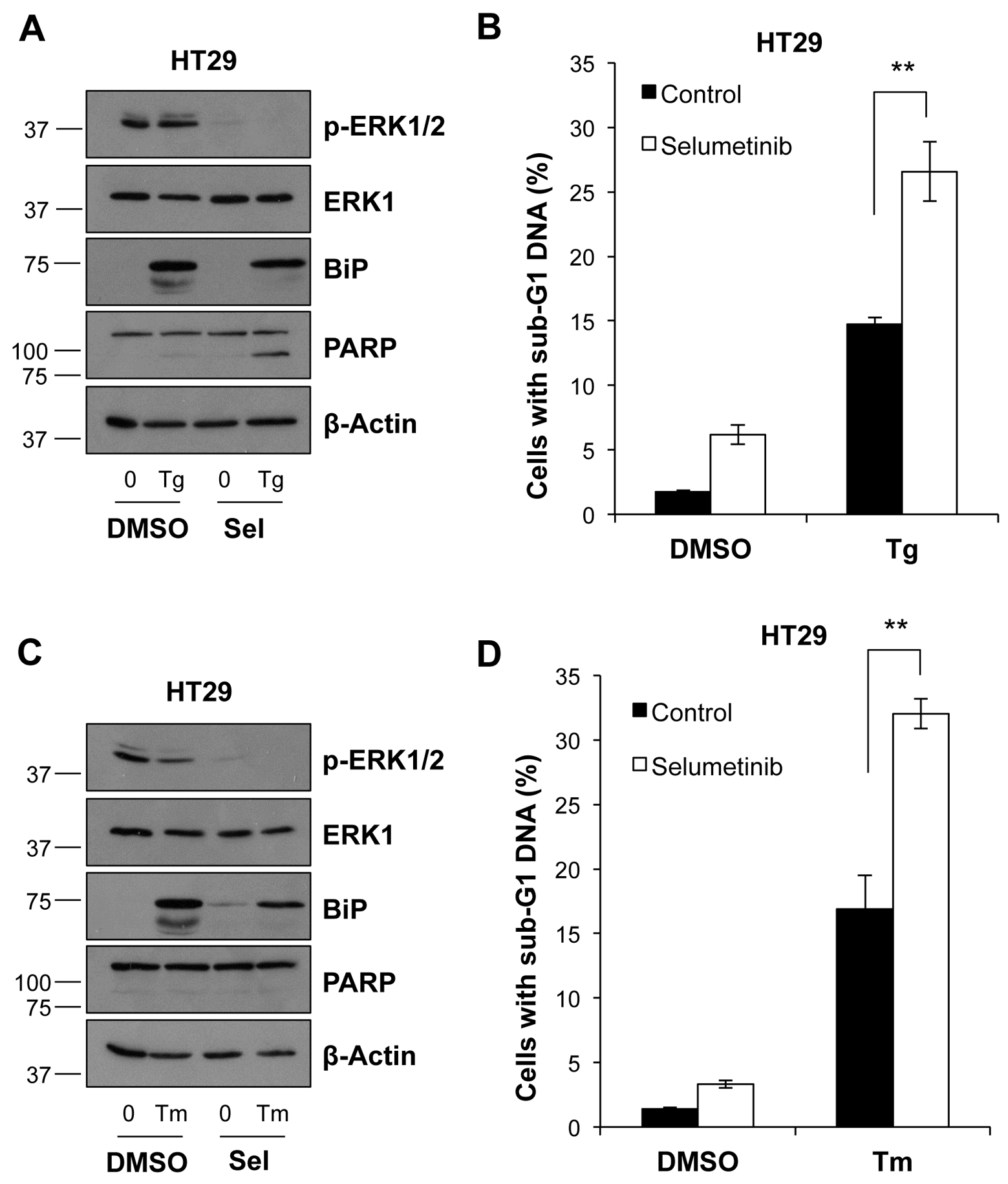

Supplement: S4 Fig — (A) HT29 cells were treated for 24 h with 1 μM Selumetinib (Sel) in addition to either DMSO or 30 nM Tg. Whole cell lysates were separated by SDS-PAGE and analysed by immunoblotting using the indicated antibodies. Results shown are representative of three independent experiments. (B) HT29 cells were treated as in (A) for 48 h, cells were fixed and stained with propidium iodide prior to analysis by flow cytometry. (C) HT29 cells were treated with 1 μM Selumetinib (Sel) and DMSO or 0.5 μg ml-1 Tm. Whole cell lysates were analysed by immunoblotting using the indicated antibodies following fractionation by SDS-PAGE. Results are representative of three independent experiments. (D) HT29 cells were treated for 48 h as described in (C), cells were fixed, stained with propidium iodide and analysed by flow cytometry. Results shown in (B) and (D) are means ± S.D. of a single experiment performed in technical triplicate and representative of three independent experiments. Statistics represent the results of Student’s unpaired t-tests; **, p < 0.01. (TIF) [file pone.0184907.s004.tif]

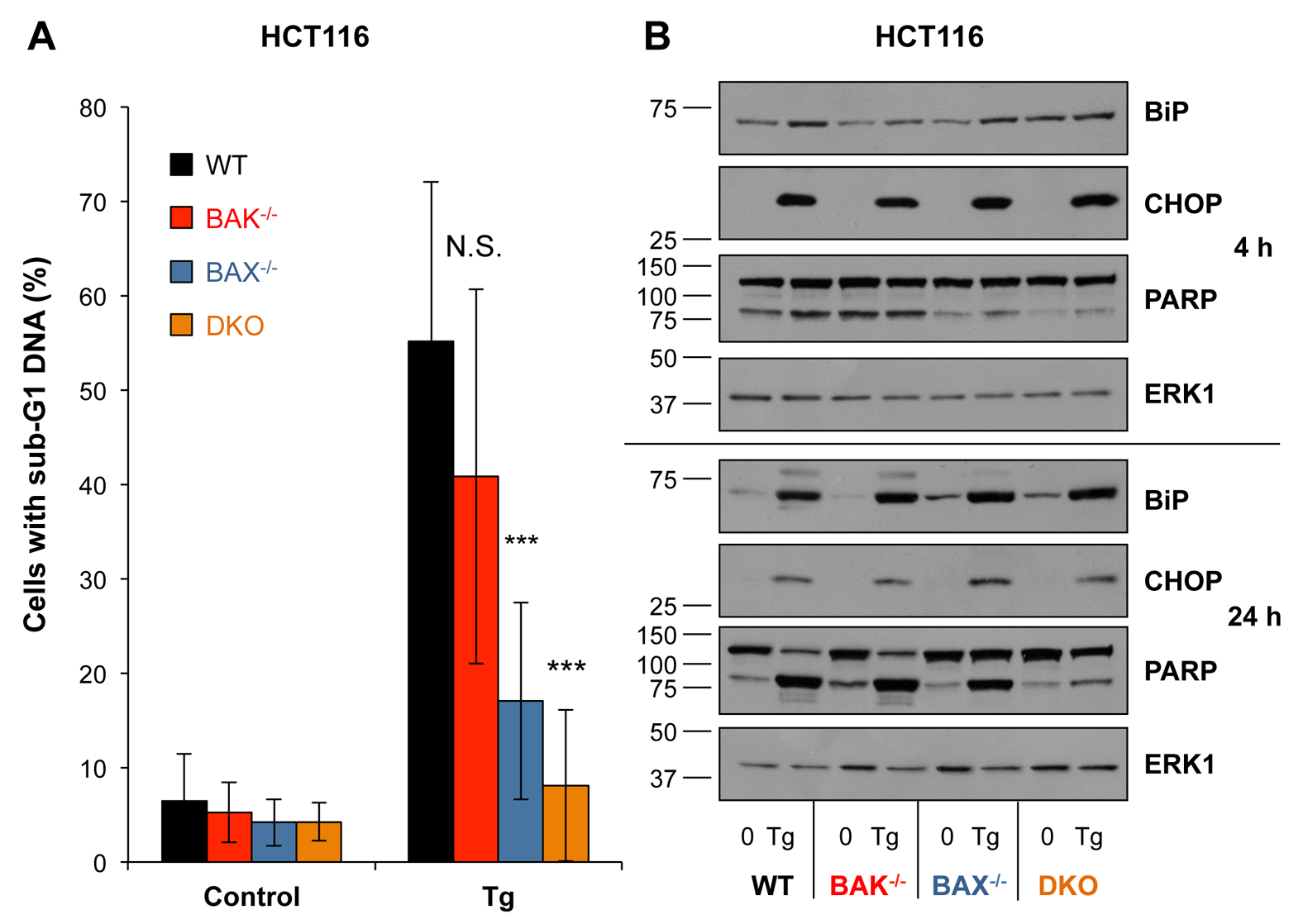

Supplement: S5 Fig — (A) HCT116, HCT116 BAK-/-, HCT116 BAX-/- or HCT116 BAK-/-, BAX-/- (DKO) cells were treated with 100 nM Tg for 48 h. Cells were fixed and analysed by flow cytometry following propidium iodide staining. Results are means ± S.D. of three independent experiments performed in technical triplicate. Statistics represent the results of two-way ANOVA and Bonferroni post-tests comparing each genotype to WT; N.S., not significant; ***, p < 0.001. (B) HCT116, HCT116 BAK-/-, HCT116 BAX-/- or HCT116 BAK-/-, BAX-/- (DKO) cells were treated with 100 nM Tg for 4 h (top panel) or 24 h (bottom panel). Whole cell lysates were separated by SDS-PAGE and analysed by immunoblotting with the indicated antibodies. Results are representative of three independent experiments. (TIF) [file pone.0184907.s005.tif]

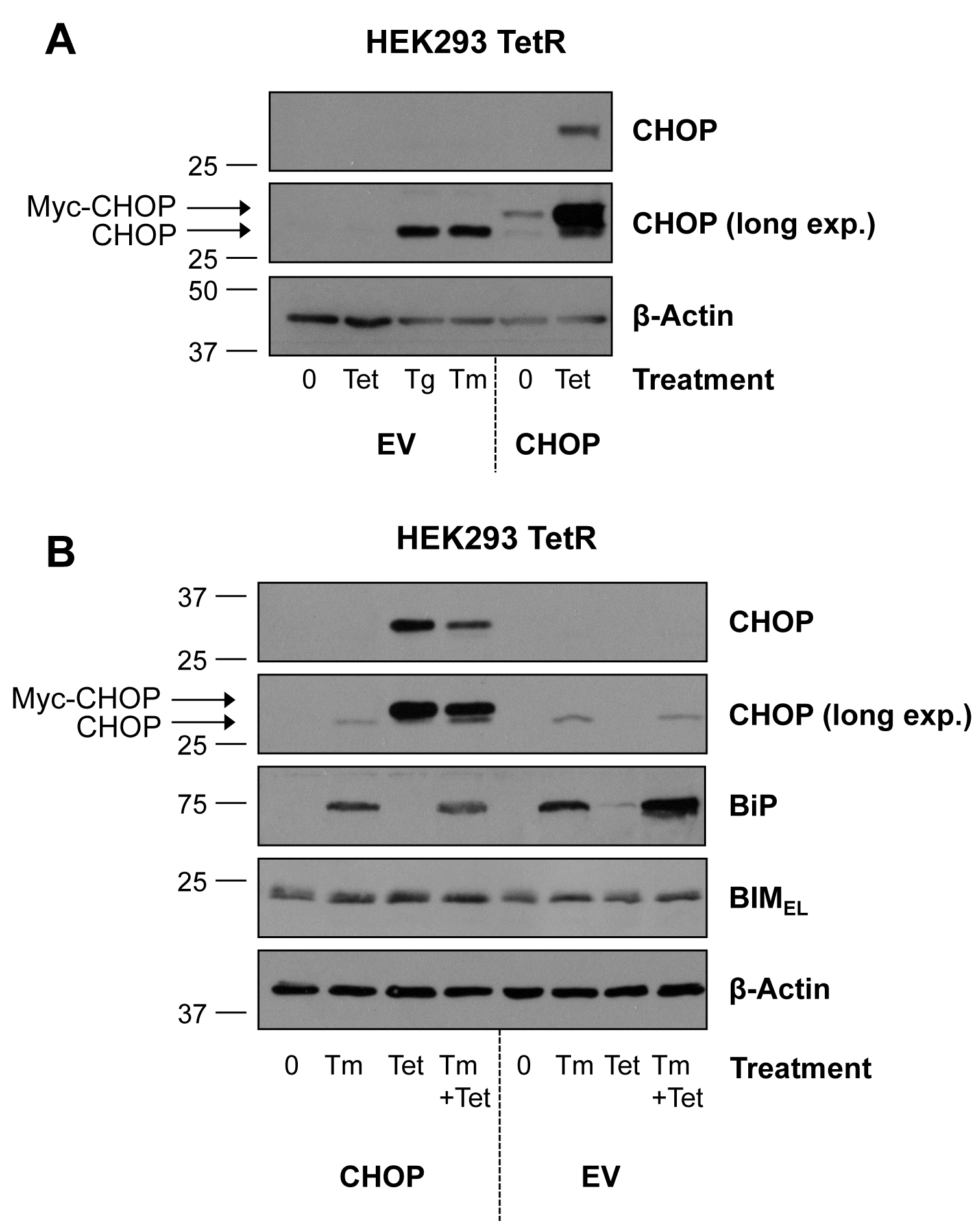

Supplement: S6 Fig — (A) HEK293 TetR cells stably transfected with pcDNA4/TO (EV) or pcDNA4/TO Myc-CHOP were treated for 4 h with 1 μg ml-1 tetracycline (Tet), 100 nM Tg or 2 μg ml-1 Tm. Whole cell lysates were analysed by immunoblotting following separation by SDS-PAGE. (B) Cell lines detailed in (A) were pre-treated for 2 h with 1 μg ml-1 tetracycline (Tet) followed by addition of 2 μg ml-1 Tm. Whole cell lysates were fractionated by SDS-PAGE and analysed by immunoblotting using the indicated antibodies. Results in (A) and (B) are from a single experiment, with comparable results detected in two inducible CHOP cell lines. (TIF) [file pone.0184907.s006.tif]

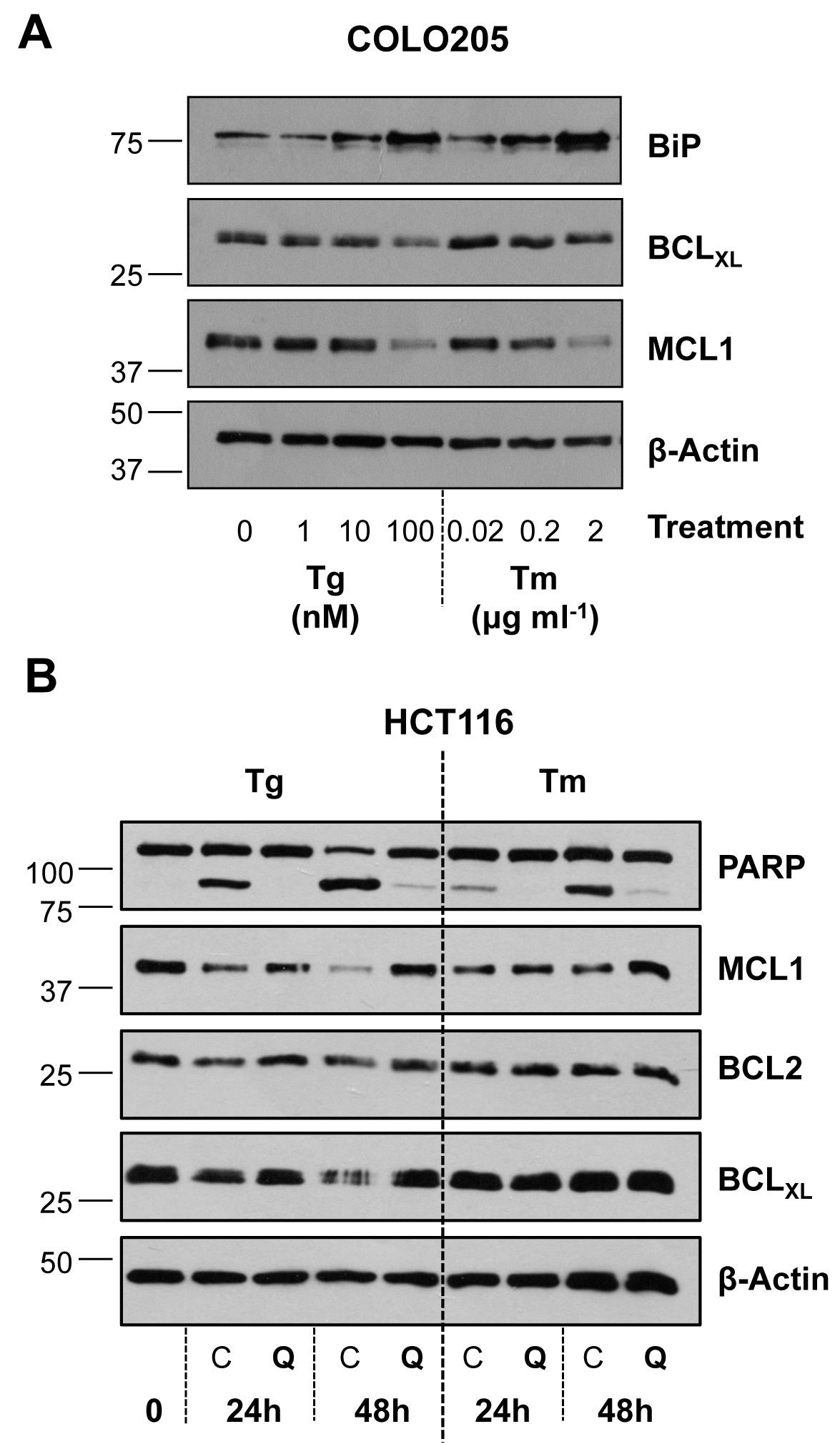

Supplement: S7 Fig — (A) COLO205 cells were treated with either Tg or Tm for 24 h. Whole cell lysates were fractionated by SDS-PAGE and analysed by immunoblotting using the indicated antibodies. Results shown are representative of three independent experiments. (B) HCT116 cells were pre-treated for 1 h with vehicle (C) or 10 μM QVD-oPH (Q) followed by 100 nM Tg or 2 μg ml-1 Tm for 24 or 48 h. Whole cell lysates were fractionated by SDS-PAGE and analysed by immunoblotting using the indicated antibodies. Results are representative of three independent experiments. (TIF) [file pone.0184907.s007.tif]

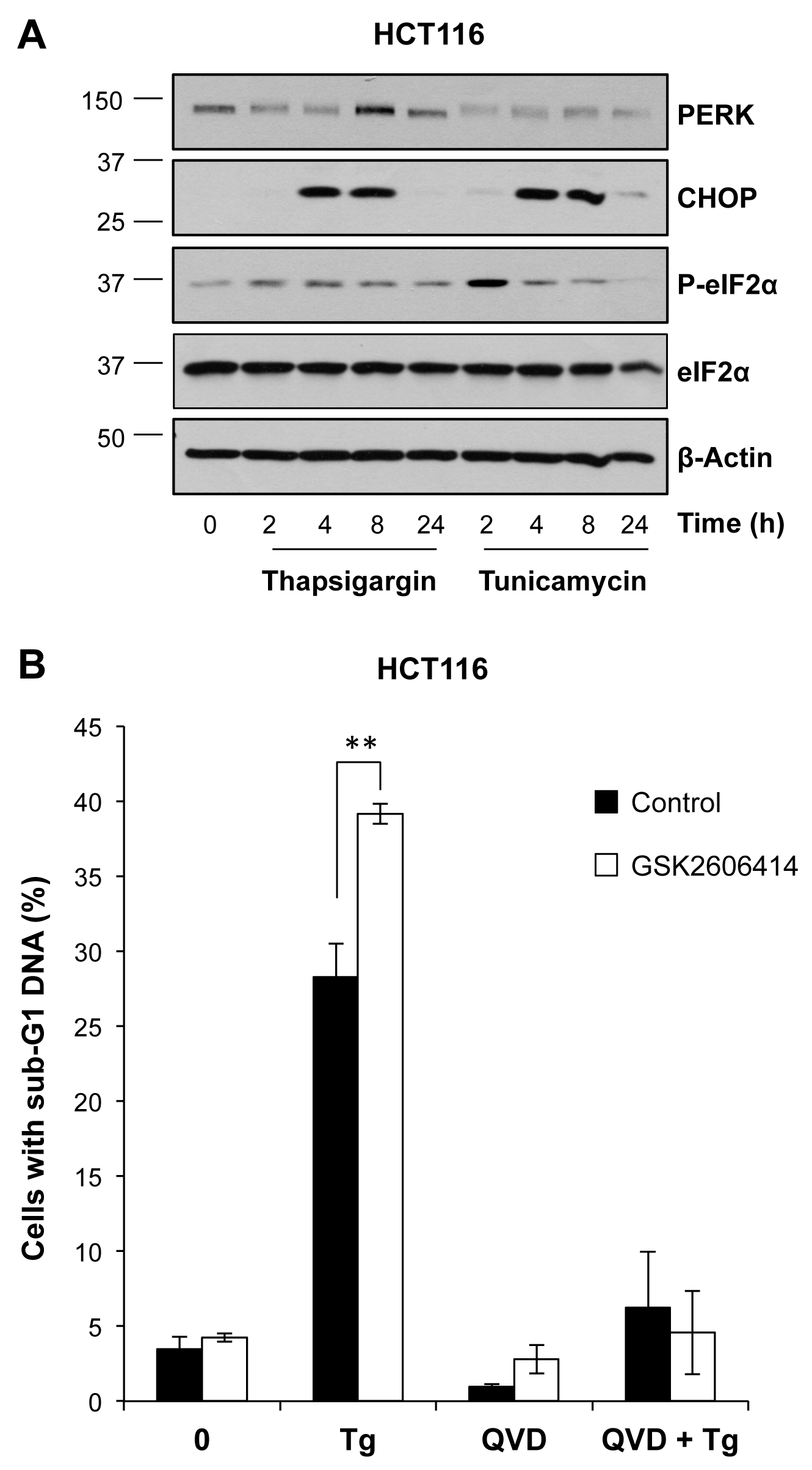

Supplement: S8 Fig — (A) HCT116 cells were treated with 100 nM Tg or 2 μg ml-1 Tm for the indicated times. Whole cell lysates were fractionated by SDS-PAGE and analysed by immunoblotting using the indicated antibodies. Results are from a single experiment. (B) HCT116 cells were pre-treated for 1 h with 100 nM GSK2606414 prior to addition of 10 nM Tg and 10 μM QVD-oPh (QVD) for 48 h. Cells were fixed and analysed by flow cytometry following propidium iodide staining. Results shown are the means ± S.D. for a single experiment, representative of two independent experiments performed in technical triplicate. Statistics represent the results of Student’s unpaired t-tests; **, p < 0.01. (TIF) [file pone.0184907.s008.tif]

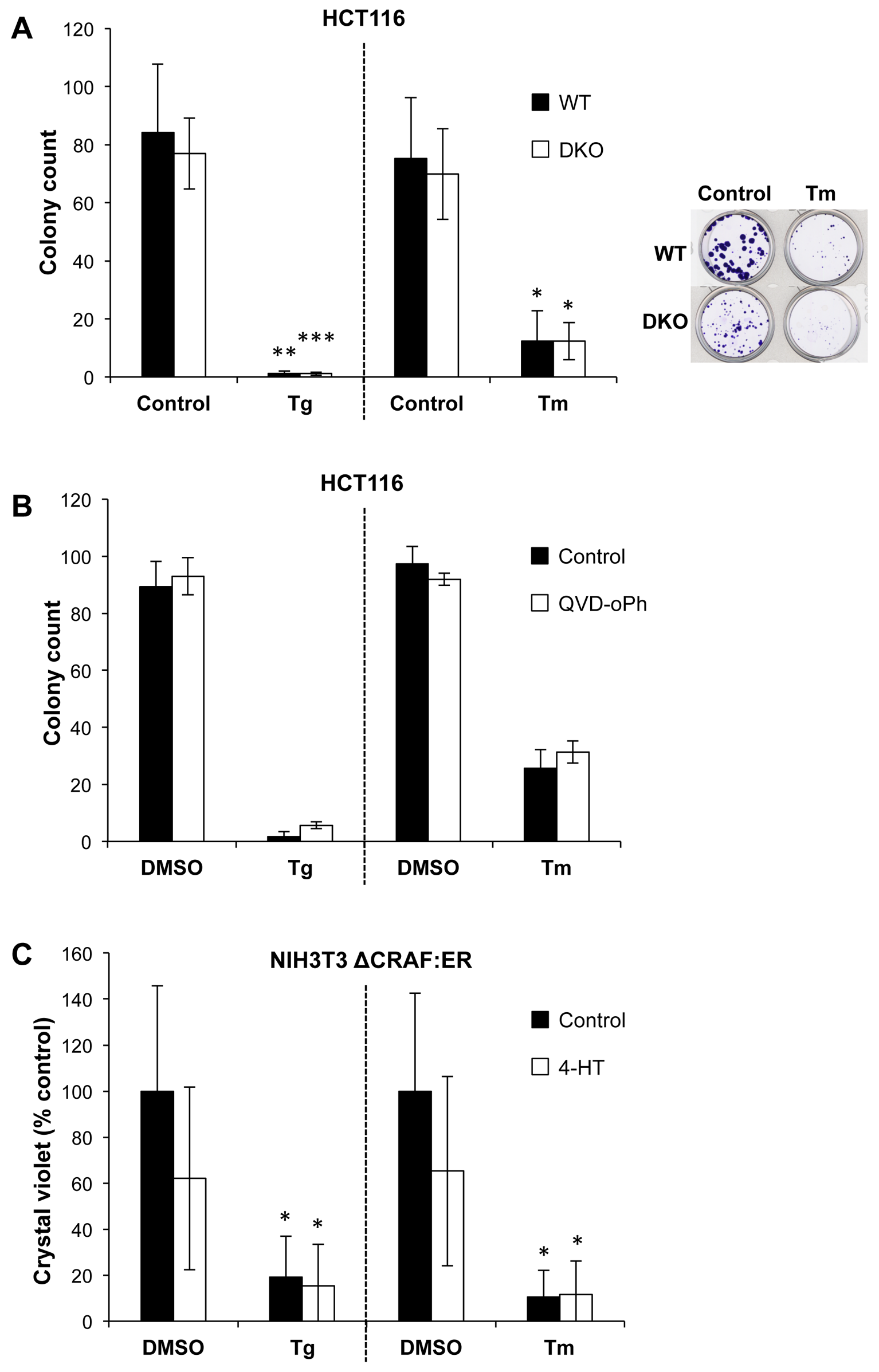

Supplement: S9 Fig — (A) HCT116 or HCT116 BAK-/-, BAX-/- (DKO) cells were treated for 72 h with 100 nM Tg (left panel) or 2 μg ml-1 Tm (right panel), treatment media was then removed and colonies were left to grow for 7 days. Cells were then fixed, stained with crystal violet and colonies with a diameter greater than approximately 0.2 mm were counted. Results shown are the combined means ± S.D. of three independent experiments performed in technical triplicate. (B) HCT116 cells were treated for 72 h with 10 μM QVD-oPh and either 100 nM Tg (left panel) or 2 μg ml-1 Tm (right panel) and colonies were analysed as in (A). Results shown are the means ± S.D. for a single experiment performed in technical triplicate. (C) NIH3T3 ΔCRAF:ER cells were pre-treated for 24 h with 100 nM 4-HT, prior to addition of 100 nM Tg (left panel) or 2 μg ml-1 Tm (right panel) for 24 h. Treatment media was removed and colonies were left to grow for 7 days before crystal violet absorbance was measured following staining. Results shown are from the combined means ± S.D. of five independent experiments performed in technical triplicate, displayed as the % staining compared to DMSO treated control and coefficient of variation. Student’s unpaired t-tests comparing colonies in the presence and absence of ER stressor in (A) and (C) are indicated as; *, p < 0.05; **, p < 0.01; ***, p < 0.001. (TIF) [file pone.0184907.s009.tif]

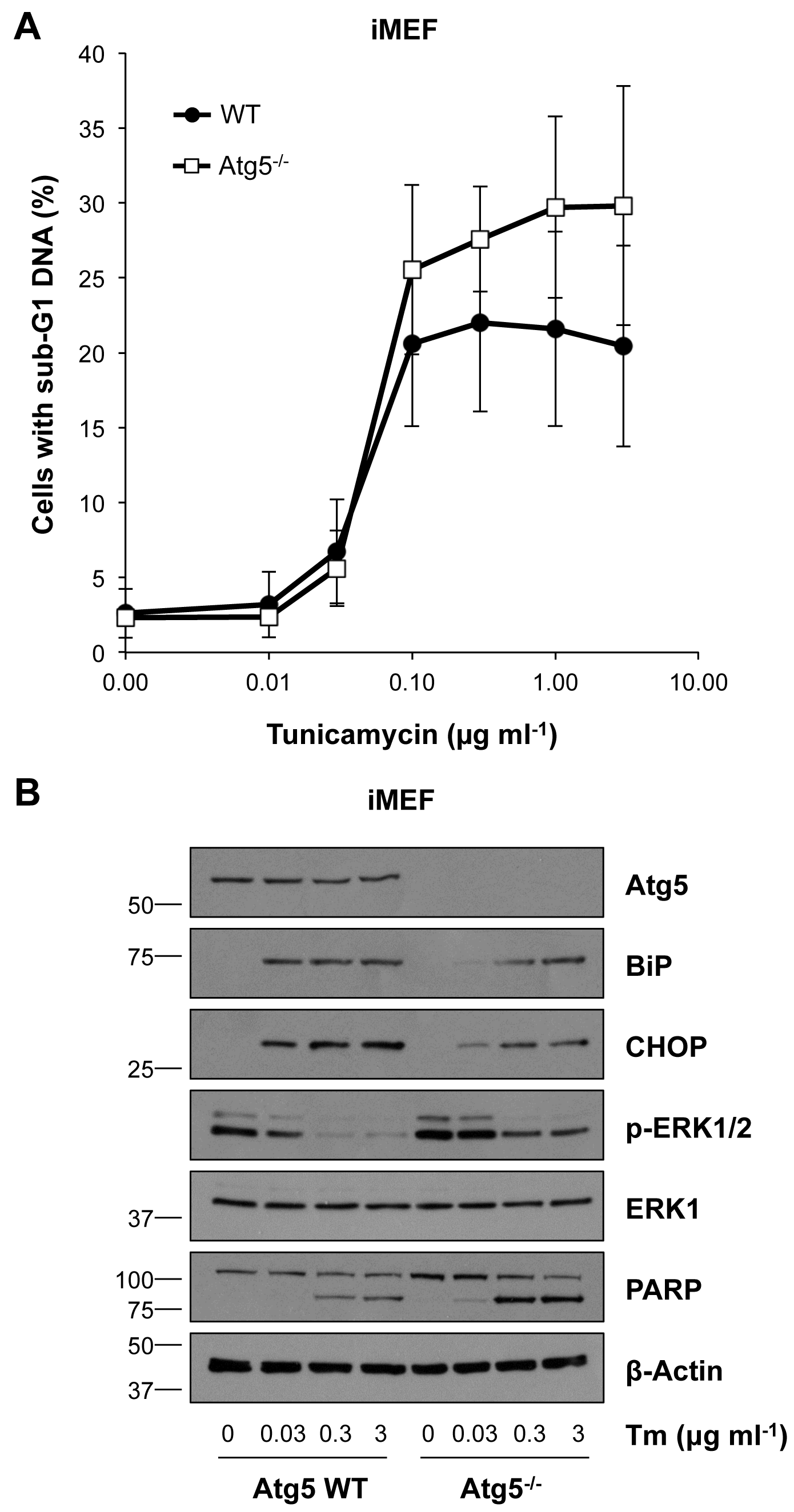

Supplement: S10 Fig — (A) Atg5 WT and Atg5-/- iMEFs were treated for 48 h with the indicated concentration of Tm. Cells were fixed and analysed by flow cytometry following propidium iodide staining. Results shown are the combined means ± S.D. of three independent experiments performed in technical triplicate. (B) Atg5 WT and Atg5-/- iMEFs were treated with the indicated concentration of Tm for 24 h. Whole cell lysates were analysed by immunoblotting using the indicated antibodies after fractionation by SDS-PAGE. Results shown are representative of three independent experiments. (TIF) [file pone.0184907.s010.tif]
